# Discovery of an evodiamine derivative for PI3K/AKT/GSK3β pathway activation and AD pathology improvement in mouse models

**DOI:** 10.3389/fnmol.2022.1025066

**Published:** 2023-01-09

**Authors:** Shuo Pang, Siyuan Li, Hanzeng Cheng, Zhuohui Luo, Xiaolong Qi, Feifei Guan, Wei Dong, Shan Gao, Ning Liu, Xiang Gao, Shuo Pan, Xu Zhang, Li Zhang, Yajun Yang, Lianfeng Zhang

**Affiliations:** ^1^Key Laboratory of Human Disease Comparative Medicine, National Health Commission of China (NHC), Institute of Laboratory Animal Science, Peking Union Medical College, Chinese Academy of Medical Sciences, Beijing, China; ^2^Beijing Engineering Research Center for Experimental Animal Models of Human Diseases, Institute of Laboratory Animal Science, Peking Union Medical College, Chinese Academy of Medical Sciences, Beijing, China; ^3^Beijing Key Laboratory of Active Substance Discovery and Drug Ability Evaluation, Institute of Material Medical, Chinese Academy of Medical Sciences and Peking Union Medical College, Beijing, China; ^4^Neuroscience Center, Chinese Academy of Medical Sciences, Beijing, China

**Keywords:** Alzheimer's disease, evodiamine, Tau hyperphosphorylation, PI3K/AKT/GSK3β pathway, Aβ pathology

## Abstract

Alzheimer's disease (AD) is a neurodegenerative disease characterized by progressive neurodegeneration and cognitive decline. Evodiamine, a main component in Chinese medicine, was found to improve cognitive impairment in AD model mice based on several intensive studies. However, evodiamine has high cytotoxicity and poor bioactivity. In this study, several evodiamine derivatives were synthesized *via* heterocyclic substitution and amide introduction and screened for cytotoxicity and antioxidant capacity. Under the same concentrations, compound **4c** was found to exhibit lower cytotoxicity and higher activity against H_2_O_2_ and amyloid β oligomers (AβOs) than evodiamine *in vitro* and significantly improve the working memory and spatial memory of 3 x Tg and APP/PS1 AD mice. Subsequent RNA sequencing and pathway enrichment analysis showed that **4c** affected AD-related genes and the AMPK and insulin signaling pathways. Furthermore, we confirmed that **4c** recovered PI3K/AKT/GSK3β/Tau dysfunction *in vivo* and *in vitro*. In conclusion, **4c** represents a potential lead compound for AD therapy based on the recovery of PI3K/AKT/GSK3β pathway dysfunction.

## Introduction

Alzheimer's disease (AD), the most common type of dementia, is a devastating illness that causes progressive neurodegenerative changes and cognitive decline. AD currently affects 43.8 million people worldwide, and the incidence of AD is expected to increase dramatically in the coming decades (GBD 2016 Dementia Collaborators., 2019). Single nucleotide polymorphisms (SNPs) or mutations in more than 100 genes have been linked to the increased risk of AD, including *App, Psen1, Psen2, Apoe, Trem2, Pon1, Aph1b, Adam10, Clu, Abca7, Slc24a4*, and *Tomm40* (Cacace et al., 2016; Nie et al., 2017; Chiba-Falek et al., 2018; Kim, 2018; De Roeck et al., 2019; Bertram and Tanzi, 2020). In addition to genetic factors, several acquired factors have been found to increase AD risk, including cerebrovascular diseases, diabetes, hypertension, obesity, dyslipidemia, neurotoxicity, depression, age, and social culture (Silva et al., 2019; Zhang et al., 2021). Despite the rapid developments in AD pathophysiology, the etiology of AD is complex and a complete understanding of the underlying pathogenic mechanisms is lacking. This limited understanding of its pathogenesis is a barrier to drug discovery, and there is currently no effective therapy for AD. Thus, efforts toward drug discovery for AD are encouraged.

**Figure 1:**
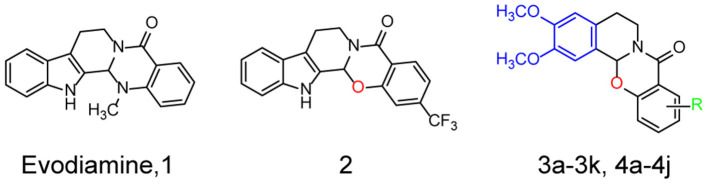
Structures and the optimization strategies of evodiamine, **2**, and the target compounds.

Recently, a number of natural compounds for improving AD symptoms were found, which suggested that natural compounds were potential resources for AD drug discovery (Wang et al., 2016). Authors of previous studies and of the present study reported that evodiamine (Evo, compound **1**, Figure 1), a quinazolinone alkaloid isolated from the fruit of *Evodiae fructus*, improves the pathological symptoms of AD by reducing acetylcholinesterase (AChE) activity, inhibiting oxidative stress, and reducing neuroinflammation (Wang et al., 2016; Zhang et al., 2018; Fang et al., 2020; Chou and Yang, 2021). However, evodiamine (Evo) exhibits high cytotoxicity and poor bioactivity (Gavaraskar et al., 2015; Pang et al., 2020). In addition, its highly active derivatives are needed to investigate its mechanism of action. In an earlier study, we reported that the cytotoxicity of evodiamine could be reduced by replacing its nitromethyl group with an oxygen atom (**2**, Figure 1), thus opening the doors for further research and development of evodiamine derivatives (Pang et al., 2020).

Herein, a series of evodiamine derivatives, compounds **3a−3k** and **4a−4j**, were designed and synthesized according to the strategy of heterocyclic substitution (Figure 1). 1,2-Dimethoxybenzene, a common structural segment in alkaloids, was used to substitute the indole ring. The oxygen atom of compound **2** was maintained and the introduction of an amide group (**R**) was investigated. The derivatives were screened by assessing their cytotoxicity against human neuroblastoma cells (SH-SY5Y) and human hepatocellular carcinoma (HepG2) cells and their anti-H_2_O_2_ activity in SH-SY5Y cells. Compared to the other derivatives and evodiamine, compound **4c** exhibited the lowest cytotoxicity and highest anti-H_2_O_2_ activity. In addition, compound **4c** prevented Aβ oligomer- and H_2_O_2_-induced cytotoxicity *in vitro* and improved AD pathology and cognitive behavior in AD mice. Compound **4c** compensated for the PI3K/AKT/GSK3β signaling pathway dysfunction in AD mice, providing insights into the molecular mechanisms of AD treatment.

## Materials and methods

### Chemistry

An Agilent Technologies LC/MSD TOF instrument was used to record the high-resolution mass spectra. Varian Mercury 400 and 100 MHz spectrometers were used to record the ^1^H and ^13^C NMR spectra. High-performance liquid chromatography (HPLC, Shimadzu LC-20A) with a reverse-phase C18 column (4.6 mm × 150 mm, 5 mm, Shim-pack VP-ODS) was used to determine the purity of the target compounds (all above 95%). The detailed chemical data, the ^1^H and ^13^C NMR spectra, and the high-resolution mass spectra are provided in the Supplementary material.

### Cell culture

SH-SY5Y and HepG2 cells were cultured in Dulbecco's modified Eagle's medium (DMEM, 11965-92, Gibco) with 10% fetal bovine serum (FBS, 10099-141C, Gibco) and 1% antibiotic penicillin-streptomycin solution (15070063, Gibco) in an incubator at 5% CO_2_ and 37°C.

### Animal treatments

APP^swe^/PS1^Δ*E*9^ double-transgenic mice (APP/PS1mice) and APP^swe^/PS1^M146V^/Tau^P301L^ triple-transgenic mice (3 x Tg mice) with a C57BL/6J genetic background were bred in our laboratory (Yuan et al., 2011). The APP/PS1 mice exhibit typical senile plaques at 4.5 months, while the 3 x Tg mice develop typical p-Tau accumulation at 8 months (Billings et al., 2005; Yuan et al., 2011; Huber et al., 2018). As described previously (Fang et al., 2019), we chose APP/PS1 mice to explore Aβ pathology and 3 x Tg mice to investigate Tau pathology. The mice experiments were approved by the Animal Care and Use Committee at the Institute of Laboratory Animal Science, Peking Union Medical College (Approval no. MYW19006).

Male and female 3 x Tg mice and C57BL/6J mice (WT) aged 9 months were randomly assigned to six groups of 12 animals. The WT mice were treated with saline (WT group) or **4c** (200 μg/kg, WT-4c-H group) and the 3 x Tg mice were treated with saline (3 x Tg group), **4c** (20 μg/kg, 4c-L group; 200 μg/kg, 4c-H group), or evodiamine (200 μg/kg, Evo group) *via* intraperitoneal injection (IP) every 2 days for 4 weeks. C57BL/6J mice and APP/PS1 mice aged 7 months were assigned to five groups of eight animals. The WT mice were treated with saline (WT group) or **4c** (200 μg/kg, WT-4c-H group) and the APP/PS1 mice were treated with saline (APP/PS1 group), **4c** (200 μg/kg, 4c-H group), or evodiamine (200 μg/kg, Evo group) by IP every 2 days for 4 weeks.

### Determination of the lethal dose 50 (LD_50_)

SH-SY5Y cells and HepaG2 cells were inoculated into 96-well plates at a density of 10,000 cells/100 μl/well in six replicates and incubated for 24 h to allow them to adhere to the plate bottom. The cells were co-incubated with different concentrations of evodiamine derivatives (0, 10^−4^, 10^−3^, 10^−2^, 10^−1^, 1, 10, 50, and 100 μg/ml) and different concentrations of evodiamine (0, 10^−4^, 10^−3^, 10^−2^, 10^−1^, 1, 5, 10, and 50 μg/ml) for 24 h. Cell viability was analyzed using a cell counting kit-8 (CCK8) assay kit (CK04-500, Dojindo). The LD_50_ was estimated from the compound concentration that resulted in 50% viability.

### Evaluation of the effect of evodiamine derivatives on H_2_O_2_ resistance

SH-SY5Y cells were inoculated into plates and incubated for 24 h to allow them to adhere to the plate bottom. The cells were then co-incubated with 75 μM H_2_O_2_ and an evodiamine derivative at a dose of 10^−2^, 10^−1^, or 1 μg/ml or **4c** at a dose of 10^−4^, 10^−3^, 10^−2^, 10^−1^, or 1 μg/ml for 24 h and cell viability was analyzed.

### Determination of cell death by calcein-AM/PI double staining

SH-SY5Y cells were inoculated into plates and incubated for 24 h to allow them to adhere to the plate bottom. The cells were then co-incubated with 75 μM H_2_O_2_ and **4c** or Evo at a dose of 10^−4^, 10^−3^, 10^−2^, 10^−1^, 0, or 1 μg/ml for 24 h. Next, calcein-AM/PI double staining was performed according to the manufacturer's instructions (C542, Dojindo). The staining solution containing 1 μM calcein-AM and 0.5 μM PI was added to the wells of the plate and incubated for 15 min in an incubator. Finally, the number of living (green) and dead cells (red) were captured using a microscope (BX53; Olympus Corporation).

### Real-time cell status assay

Aβ1–42 oligomers (AβOs) were obtained from Chinapeptides (04010011526, Chinapeptides). The synthesized monomeric peptides were dissolved in cooled 1,1,1,3,3,3-hexafluoro-2-propanol (HFIP) and incubated for 60 min at room temperature, followed by placing the peptide–HFIP solution on ice for 10 min and drying at room temperature. The peptide was added to DMSO to form a peptide film, diluted with Ham's F12-free medium to a final concentration of 50 μM, incubated for 24 h at 4°C, and centrifuged at 14,000 rpm for 10 min, with the supernatant being the Aβ oligomers. Finally, it is freeze-dried to a powder. As previously reported (Rodriguez-Garcia et al., 2021), the effects of **4c** and evodiamine on AβOs cytotoxicity were detected by a real-time cell analyzer (RTCA-DP, ACEA Biosciences Inc, USA). SH-SY5Y cells were inoculated in E-plates (ACEABiosciences, 00300600890) and cultured for 3 h to allow cell attachment. The medium was then replaced with another medium containing 25 μM AβO alone, 25 μM AβOs with 0.1 μg/ml **4c**, or 25 μM AβOs with 0.1 μg/ml evodiamine, respectively. The plates were placed in a real-time cell analyzer under a CO_2_ incubator at 37°C for 200 h. The cell index (CI) was determined by measuring the cell-to-electrode responses of the cells attached to the E-plates, which represented the cell viability status and cell number. The CI was monitored every 15 min during the 200-h experiment time.

### Western blotting

To detect AβOs by western blotting, brain tissues were homogenized in a TBS extraction buffer comprising protease inhibitor (87785, Thermo Fisher Scientific), 1 mM NaF, 50 mM Tris pH 7.4, phosphatase inhibitor cocktail (78420, Thermo Fisher Scientific), 150 mM NaCl, 1 mM NaVO_3_, and 1 mM phenylmethylsulfonylfluoride (PMSF, 36978, ThermoFisher Scientific) as reported by Zhang et al. (2017).

Western blotting was performed as previously described (Pang et al., 2021). The brain tissue homogenates were separated by SDS–PAGE and electroeluted onto nitrocellulose membranes (Immobilon NC; Millipore). The membranes were initially incubated with primary antibodies overnight (Table 1) followed by HRP-linked secondary antibodies. Finally, the proteins were visualized and analyzed. The antibodies and working dilutions are summarized in Table 1.

**Table 1:** Antibodies used in this study.

**Primary antibodies**	**Source**	**Catalog no**.	**WB**	**IHC**	**IF**
6E10	Biolegend	803001	1:500	—	1:150
IBA1	CST	17198	—	—	1:150
GFAP	Abcam	ab7260	—	—	1:200
T231 (p-TAU at Thr231)	Abcam	ab151559	—	1:200	—
T181 (p-TAU at Thr181)	Abcam	ab75679	1:500	1:200	1:100
AT8 (p-TAU at Ser202/Thr205)	Thermo Fisher	MN1020	1:500	—	1:100
p-PI3K	CST	4228	1:500	—	—
PI3K	CST	4292S	1:500	—	—
p-AKT (Ser473)	CST	9271	1:1,000	—	—
AKT	CST	4691s	1:1000	—	—
p-GSK3α/β (Ser21/9)	CST	9331s	1:1,000	—	—
GSK3β	CST	9315	1:1,000	—	—
GAPDH	Abcam	ab201822	1:10,000	—	—
TAU5	Millipore	577801	1:500	—	—
488 goat anti-mouse IgG	Invitrogen	A11029	—	—	1:200
555 goat anti-rabbit IgG	Invitrogen	A21429	—	—	1:200
HRP-anti-mouse IgG	ZSGB-BIO	PV9002	—	1:20	—
HRP-t anti-rabbit-IgG	ZSGB-BIO	PV9001	—	1:20	—

### Pathological assay

Mouse brain tissues were fixed, dehydrated, and embedded in paraffin and the sections were dewaxed according to a standard procedure. The sections were blocked in 1% BSA for 30 min and then incubated with primary antibodies (Table 1). Then, the sections were applied to immunofluorescent or immunohistochemical staining. For immunofluorescent staining, the sections were incubated with fluorescent secondary antibodies (Table 1) in a dark box for 1 h, washed with phosphate-buffered saline (PBS), and mounted with DAPI buffer (ZLI-9557, ZSGB BIO). For immunohistochemical staining, the sections were incubated with HRP-labeled secondary antibodies, washed with PBS, visualized with DAB peroxidase substrate, and mounted in neutral gum. The sections were then scanned under a digital slide scanner (Pannoramic250 FLASH, 3DHISTECH). Image quantification was performed using ImageJ as previously described (Luo et al., 2020).

### Cognitive behavior analysis

After 1 month of treatment, the mice were subjected to the *Y* maze test according to the reported methods (Spangenberg et al., 2019). One mouse was placed in the *Y* maze for 5 min and its activity was recorded and analyzed by an Ethovision XT system (Noldus Ltd). The percentage of alternations among the three arms was calculated as actual alternations/maximum alternations × 100.

After the *Y* maze test, the mice were subjected to the Morris water maze (MWM) test according to the reported methods (Pang et al., 2020). For five consecutive days, the mice were trained to look for the platform. The latency in looking for the hidden platform was recorded. On day 6, the mice were placed in a heterolateral quadrant and allowed to explore freely for 60 s in the absence of the platform. During this time, the number of crossings to the previous platform region and target quadrant occupancy were recorded. A video-tracking system was used for recording and analysis.

### Quantitative real-time PCR (qRT-PCR)

RNA was extracted using TRIzol reagent (15596018, Invitrogen) and used as the template for first-strand cDNA synthesis using a reverse transcription kit (RR047A, TaKaRa). Next, the cDNA was used for qRT-PCR using a QuanStudio™3 Real-Time PCR instrument (Thermo Fisher Scientific) with SYBR Green real-time PCR kits (RR820A, TaKaRa). We detected the mRNA expressions of *Adam10, Snca, Caspase7, Ide*, and *Aph-1b*; *Gapdh* expression was used for normalization. The primers for qRT-PCR are summarized in Table 2.

**Table 2:** Primers used in this study.

**Gene**	**Forward sequences**	**Reverse sequences**
*Adam10*	5′-CTCGTCGGGACCCAGC-3′	5′-AAAGGATTTCCATACTGACCTCCC-3′
*Snca*	5′-AGCCTGTGCATCTATCTGCG-3′	5′-TTGCTCCACACTTTCCGACTT-3′
*Caspase7*	5′-CCTCTGGGACTTTTGCTTTCAG-3′	5′-TCATCGGTCATCGTTCCCA-3′
*Ide*	5′-GACCACGAGGCTATACGTCC-3′	5′-ATTGCCACCCGCACATTTTC-3′
*Aph-1b*	5′-TGTTGGCCTATGTTTCTGGCT-3′	5′-CAAAGAACACAACGCCCCAG-3′
*Gapdh*	5′-GGGTTCCTATAAATACGGACTGC-3	5′- CAATACGGCCAAATCCGTTCA-3

### RNA sequencing analysis

Mice treated with or without **4c** were sacrificed and the hippocampal tissues were dissected. RNA isolation, library preparation, Illumina RNA sequencing, and data processing were performed as previously described (Lu et al., 2018). Genes with RPKM <1 were excluded and genes with a |fold change| >1 and a *P*-value of < 0.05 were considered differentially expressed.

### Statistical analysis

We analyzed the data by two-tailed unpaired *t*-tests and a one-way ANOVA followed by Tukey's *post hoc* analysis. Data are presented as the mean ± SEM. A *P*-value of < 0.05 indicated a statistically significant difference.

## Results

### Synthesis of evodiamine derivatives

**Scheme 1:**
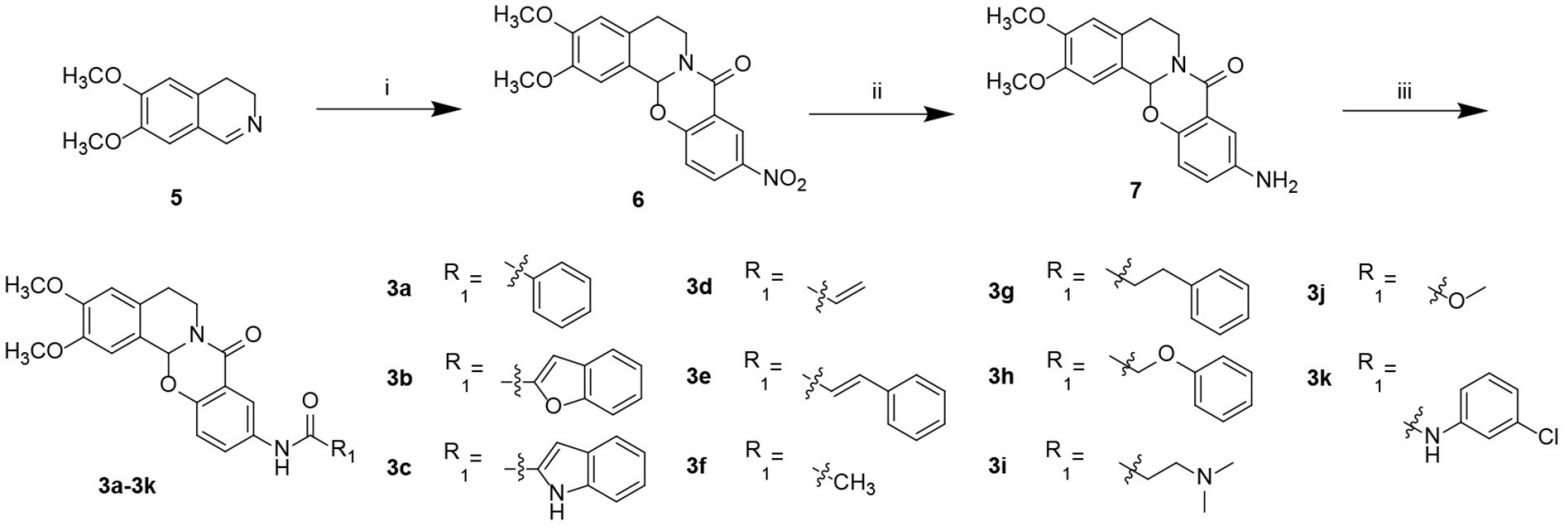
Preparation of target compounds **3a**–**k**. Reagents and conditions: (i) 5-nitrosalicylic acid, 1-(3-dimethylaminopropyl)-3-ethylcarbodiimide hydrochloride, dichloromethane, rt; (ii) 10% Pd/C, hydrogen, methanol, and rt; (iii) 2-(7-azabenzotriazol-1-yl)-N,N,N′,N′-tetramethyluronium hexafluorophosphate, N,N-diisopropylethylamine, dichloromethane, and rt.

**Scheme 2:**
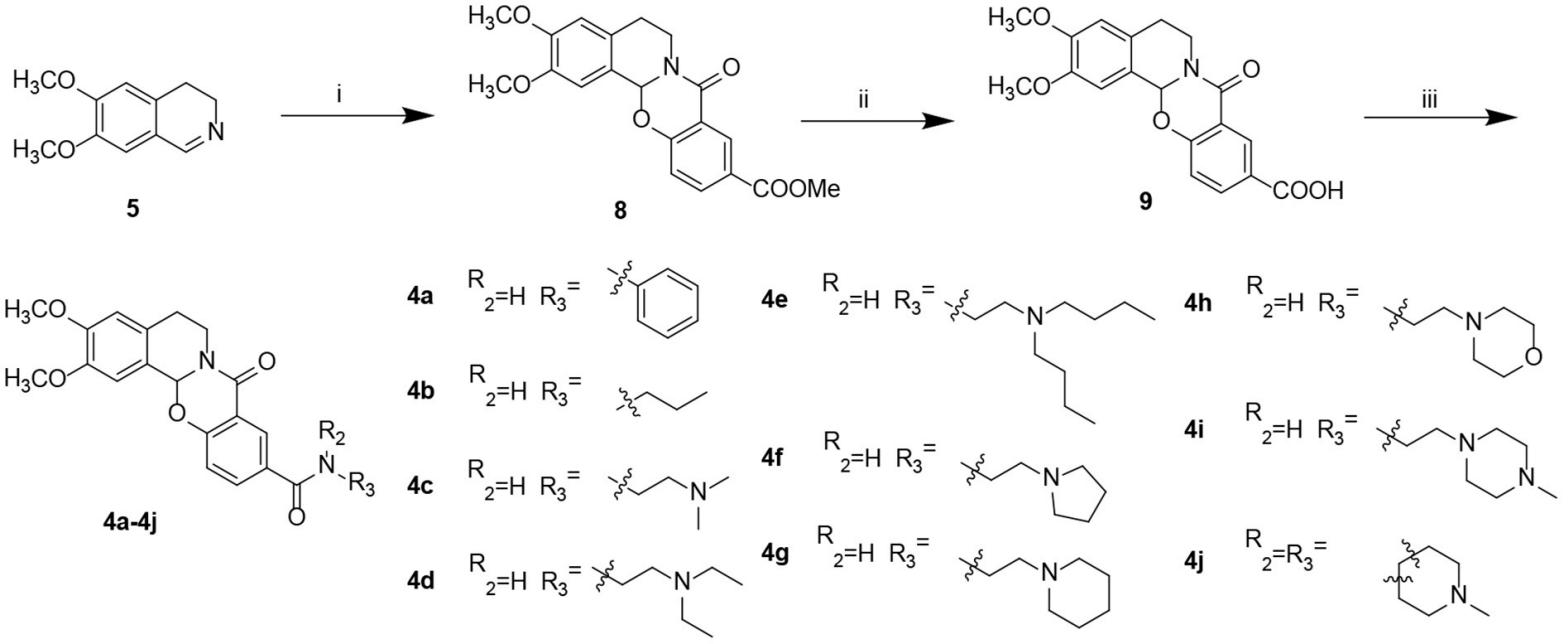
Preparation of target compounds **4a**–**j**. Reagents and conditions: (i) 2-hydroxy-5-(methoxycarbonyl) benzoic acid, 1-(3-dimethylaminopropyl)-3-ethylcarbodiimide hydrochloride, dichloromethane, and rt; (ii) LiOH, methanol: H_2_O, and rt; and (iii) 2-(7-azabenzotriazol-1-yl)-N,N,N′,N′-tetramethyluronium hexafluorophosphate, N,N-diisopropylethylamine, dichloromethane, and rt.

Starting from 6,7-dimethoxy-3,4-dihydroisoquinoline (**5**, Scheme 1), 5-nitrosalicylic acid was treated with 1-(3-dimethylaminopropyl)-3-ethylcarbodiimide hydrochloride in dichloromethane to give intermediate **6** (Yang et al., 2016). Reduction of **6** by 10% Pd/C under hydrogen resulted in **7**. Target compounds **3a–k** were furnished by the condensation of **7** with different acids, acyl chlorides, and isocyanates. The synthesis route for exchanging amides to obtain compounds **4a–j** is depicted in Scheme 2. Intermediate **8** was prepared from dihydroisoquinoline **5** and 2-hydroxy-5-(methoxycarbonyl) benzoic acid, followed by hydrolysis to afford **9**. Finally, compounds **4a–j** were furnished by the condensation of acid **9** and different amines.

### Selection of evodiamine derivatives based on cytotoxicity and anti-H_2_O_2_ activity

**Figure 2:**
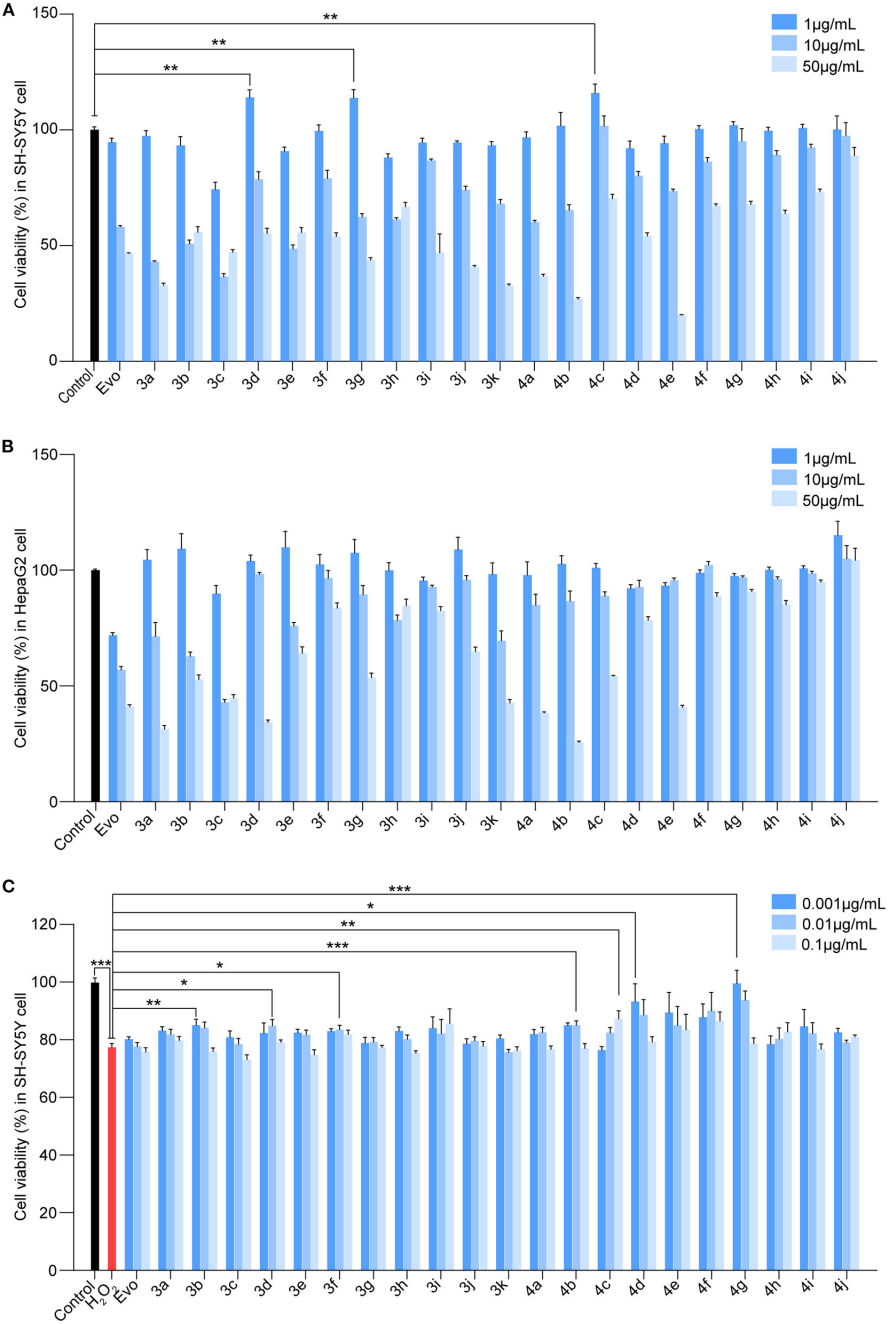
Screening for the cytotoxicity and the anti-H_2_O_2_ activity of the evodiamine derivatives. **(A, B)** SH-SY5Y cells and HepaG2 cells were treated with Evo and Evo derivatives at concentrations of 1, 10, and 50 μg/ml for 24 h. The cytotoxicity of Evo and its derivatives was subsequently assessed by detecting cell viability using CCK8. **(C)** SH-SY5Y cells were co-incubated with 75 μM H_2_O_2_ and different concentrations of Evo derivatives (10^−3^, 10^−2^, and 10^−1^ μg/ml) for 24 h, and cell viability was quantified using CCK8. Data are represented as mean ± SEM, *n* = 6; ^*^P < 0.05, ^**^P < 0.01, and ^***^P < 0.001 vs. control.

To identify Evo derivatives with lower cytotoxicity and higher anti-H_2_O_2_ activity, the cytotoxicity of the derivatives and Evo in SH-SY5Y and HepaG2 cells was evaluated by measuring cell viability. Although several compounds exhibited lower cytotoxicity than Evo and the other derivatives (Figures 2A, B, *n* = 6), compounds **3d**, **3g**, and **4c** significantly promoted SH-SY5Y cell proliferation (Figure 2A, *n* = 6; ^**^P < 0.01). Furthermore, the anti-H_2_O_2_ activity of the derivatives and Evo in SH-SY5Y cells was assessed by measuring cell viability in the presence of H_2_O_2_. Compounds **3b**, **3d**, **3f**, **4b**, **4c**, **4d**, and **4g** had higher anti-H_2_O_2_ activity than Evo and the other derivatives (Figure 2C, *n* = 6; ^*^P < 0.05, ^**^P < 0.01, and ^***^P < 0.001). Among all derivatives, **4c** had the lowest cytotoxicity, the highest anti-H_2_O_2_ activity, and the greatest neuronal cell-proliferation-promoting effect and hence was selected for further study.

### Compound 4c has a higher LD_50_ and activity against AβOs than evodiamine *in vitro*

**Figure 3:**
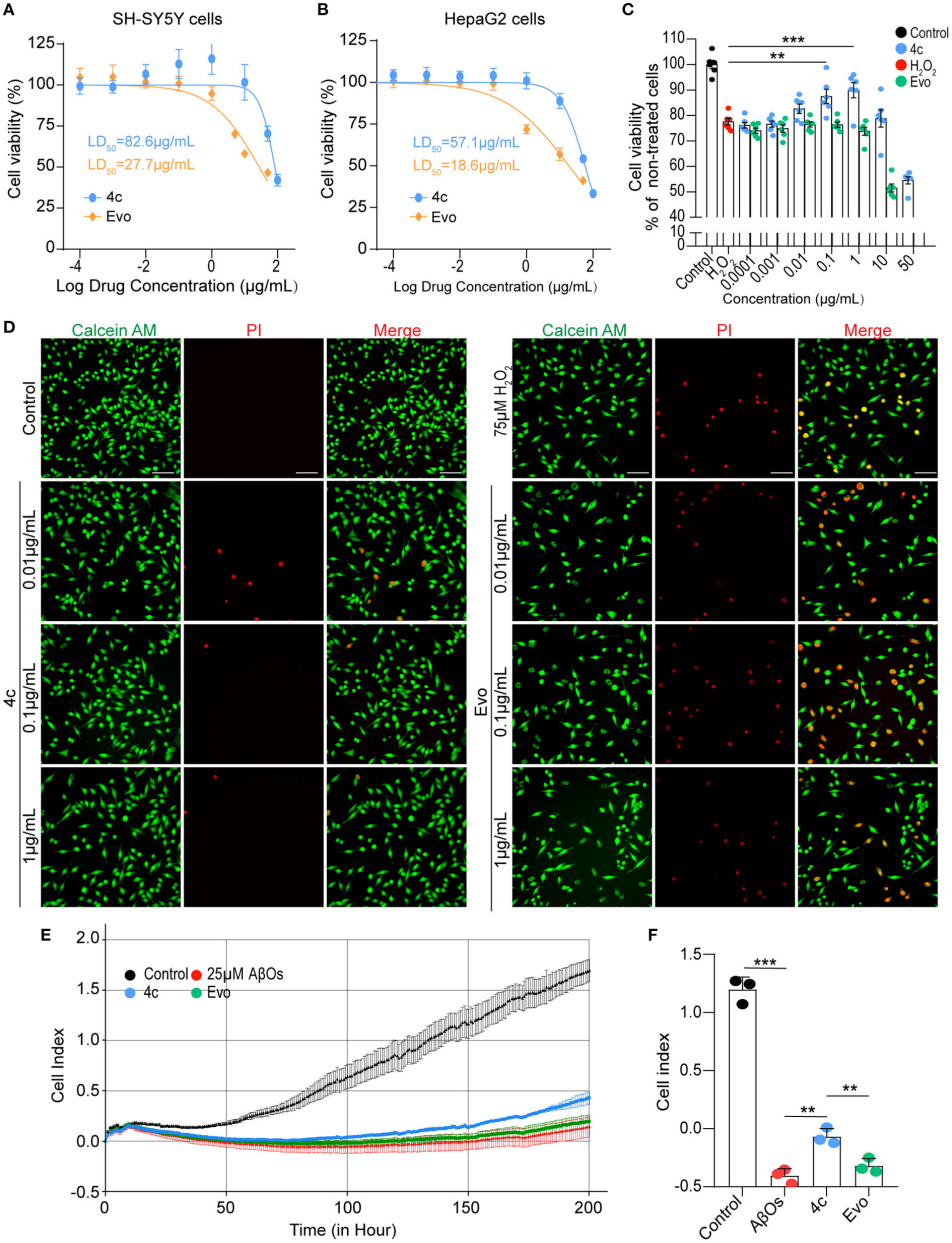
Determination of the LD_50_ of **4c** and its effects against H_2_O_2_ and AβOs. **(A, B)** The viability of SH-SY5Y and HepaG2 cells was determined by CCK8 after 24 h of exposure to different concentrations of **4c** (10^−4^, 10^−3^, 10^−2^, 10^−1^, 1, 10, 50, and 100 μg/ml) and Evo (10^−4^, 10^−3^, 10^−2^, 10^−1^, 1, 5, 10, and 50 μg/ml). The LD_50_ values of **4c** and Evo were estimated from the concentrations that resulted in 50% cell viability compared with the vehicle control. Data are represented as mean ± SEM, *n* = 6. **(C)** SH-SY5Y cells were incubated with 75 μM H_2_O_2_ alone or co-incubated with 75 μM H_2_O_2_ and different concentrations of **4c** or Evo (10^−4^, 10^−3^, 10^−2^, 10^−1^, 1, 10, and 50 μg/ml) for 24 h. Cell viability was subsequently quantified using CCK8 and normalized to the cell viability of the vehicle control. Data are represented as mean ± SEM, *n* = 6. **(D)** SH-SY5Y cells were incubated with 75 μM H_2_O_2_ alone and co-incubated with 75 μM H_2_O_2_ and different concentrations of **4c** or Evo (10^−2^, 10^−1^, and 1 μg/ml) for 24 h. The cells were then stained with calcein-AM/PI, and living and dead cells were detected by green or red fluorescence, respectively. *n* = 3, Scale bar = 100 μm. **(E)** SH-SY5Y cells were co-incubated with 25 μM AβOs without or with 0.1 μg/ml **4c** or Evo in a real-time cell analyzer for 200 h. The cell index, which represents the real-time cell status, was monitored by the analyzer and analyzed by the RTCA software. **(F)** The cell index of **4c** at 200 h was significantly higher than that of Evo. *n* = 3; ^**^P < 0.01, and ^***^P < 0.001.

The median lethal doses (LD_50_) of **4c** and Evo in SH-SY5Y and HepaG2 cells were comparatively analyzed. The LD_50_ of **4c** and Evo were 82.6 and 27.7 μg/ml, respectively, in SH-SY5Y cells (Figure 3A) and 57.1 and 18.6 μg/ml, respectively, in HepaG2 cells (Figure 3B). Thus, the LD_50_ of **4c** in SH-SY5Y and HepaG2 cells was approximately two-fold higher than that of Evo. Next, the effective concentration of compound **4c** against H_2_O_2_ in SH-SY5Y cells was determined by measuring the viability of cells treated with 10^−4^, 10^−3^, 10^−2^, 10^−1^, 1, or 10, or 50μg/ml **4c**. The minimal and optimum effective concentrations of compound **4c** against H_2_O_2_ in SH-SY5Y cells were 0.01 and 1 μg/ml, respectively (Figure 3C). The induction of cell death by H_2_O_2_ was assessed by calcein-AM/PI double staining, which showed that **4c** protected SH-SY5Y cells from cell death at concentrations of 0.01, 0.1, and 1 μg/ml, whereas Evo had no apparent protective effect against cell death (Figure 3D). Furthermore, AβO cytotoxicity was evaluated using a real-time cell analyzer that monitors cell proliferation and viability status in real time. The results showed that compound **4c** significantly reduced the AβOs cytotoxicity in SH-SY5Y cells (Figures 3E, F, *n* = 3; ^**^P < 0.01 and ^***^P < 0.001).

### Compound 4c improves working memory and spatial memory in 3 x Tg mice

**Figure 4:**
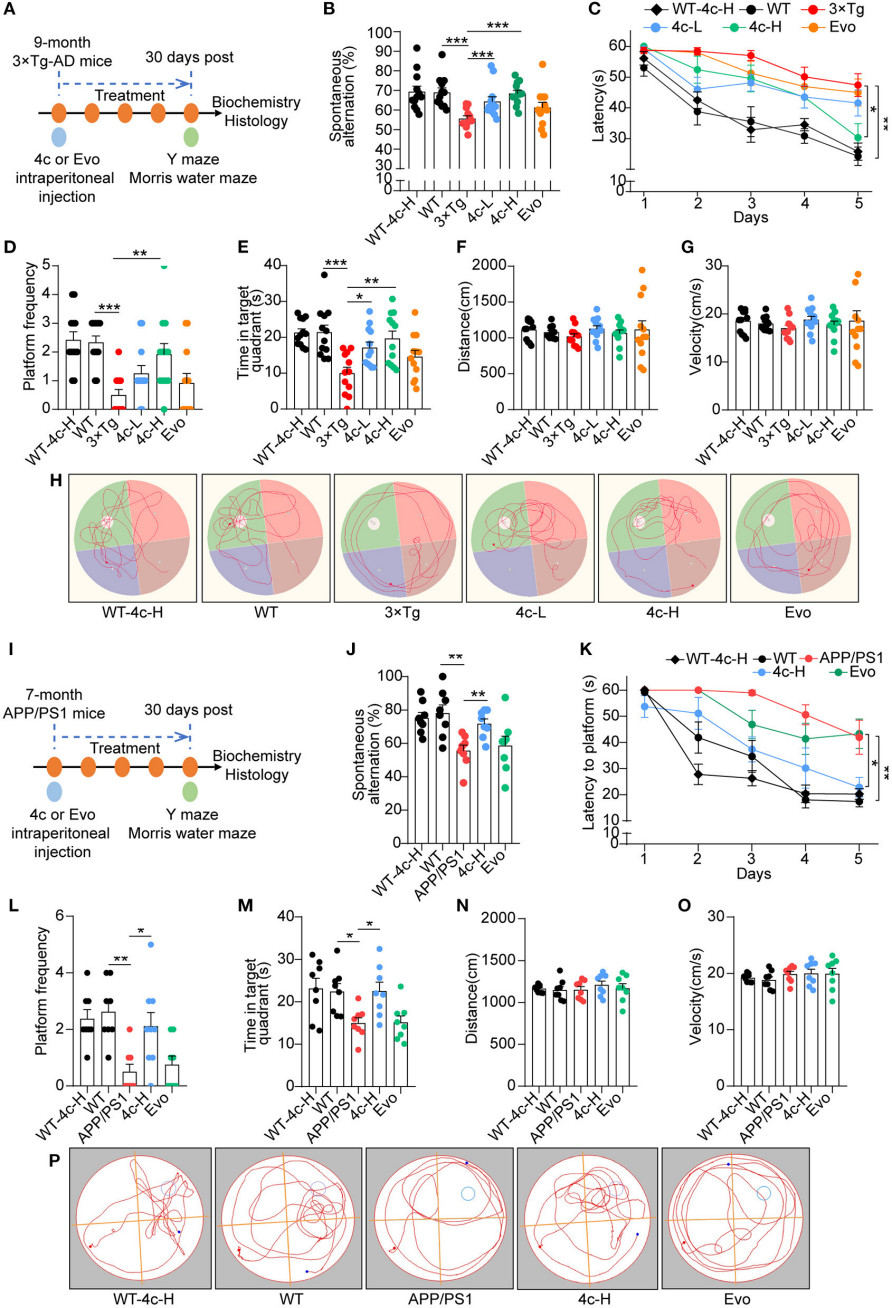
Analysis of the cognitive behavior of 3 x Tg and APP/PS1 AD mice treated with **4c**. **(A)** Schematic diagram of the experimental design for treating 3 x Tg-AD mice with **4c**. Six groups of mice were treated every 2 days for 4 weeks: WT with 200 μg/kg **4c** (WT-4c-H), WT with vehicle (WT), 3 x Tg with vehicle (3 x Tg), 3 x Tg with 20 μg/kg **4c** (4c-L), 3 x Tg with 200 μg/kg **4c** (4c-H), and 3 x Tg with 200 μg/kg Evo (Evo). **(B)** After treatment, all mice were subjected to the *Y* maze test and the percentage of alternations was calculated. **(C)** After the *Y* maze test, the mice were subjected to the MWM test and the latency to find the hidden platform during the 5-day training period was comparatively analyzed. **(D, E)** The number of platform crossings and time in the target quadrant during the probe trial on day 6 were recorded. **(F–H)** The distance, velocity, and track examples of the mice in the MWM test were recorded during the probe trial on day 6. Data are represented as mean ± SEM, *n* = 12; ^*^P < 0.05, ^**^P < 0.01, and ^***^P < 0.001. **(I)** Schematic diagram of the experimental design for treating APP/PS1 AD mice with **4c**. Five groups of mice were treated every 2 days for 1 month: WT with 200 μg/kg **4c (**WT-4c-H), WT with vehicle (WT), APP/PS1 with vehicle (APP/PS1), APP/PS1 with 200 μg/kg **4c** (4c-H), and APP/PS1 with 200 μg/kg Evo (Evo). **(J)** After treatment, working memory was assessed by performing the *Y* maze test and analyzing the percentage of alternations. **(K–M)** The treated mice were subjected to the MWM test and the latency to find the hidden platform during the 5-day training period was recorded. On day 6, the spatial memory of the mice was evaluated by monitoring the number of platform crossings and time in the target quadrant. **(N–P)** The distance, velocity, and track examples of the mice in the MWM were recorded on day 6. Data are represented as mean ± SEM, *n* = 10; ^*^P < 0.05 and ^**^P < 0.01.

We evaluated the effects of compound **4c** on cognitive decline and Tau-dependent pathology in 3 x Tg mice, which develop more typical Tau phosphorylation than APP/PS1 AD mice (Billings et al., 2005; Yuan et al., 2011; Huber et al., 2018; Fang et al., 2019). Nine-month-old 3 x Tg mice were treated with or without **4c** or Evo for 4 weeks (Figure 4A). The *Y*-maze tests showed that compound **4c** significantly improved the working memory of 3 x Tg mice in a dose-dependent manner, with increases in the number of spontaneous alternations of 15.6 and 23.1% in a dose-dependent manner (Figure 4B, *n* = 12, ^***^P < 0.001). In the MWM test, treatment with 20 or 200 μg/kg showed that compound **4c** significantly decreased the latent time before exploring the hidden platform, increased the number of platform crossings by 150 and 283.4%, respectively, and increased the target quadrant duration by 71.3 and 97.2%, respectively (Figures 4C–E, *n* = 12; ^*^P < 0.05, ^**^P < 0.01, and ^***^P < 0.001). These results showed that compound **4c** significantly improved the spatial memory of 3 x Tg mice in a dose-dependent manner. In 3 x Tg AD mice, Evo showed a tendency to improve the working and spatial memories (Figures 4B, E, *n* = 12). Compound **4c** did not alter the cognitive behavior of WT mice in the *Y*-maze and MWM tests (Figures 4B, E, *n* = 12), and neither **4c** nor Evo affected the swimming speed and distance of the WT and AD mice (Figures 4F, G, *n* = 12).

### Compound 4c inhibits Tau hyperphosphorylation in 3 x Tg mice

**Figure 5:**
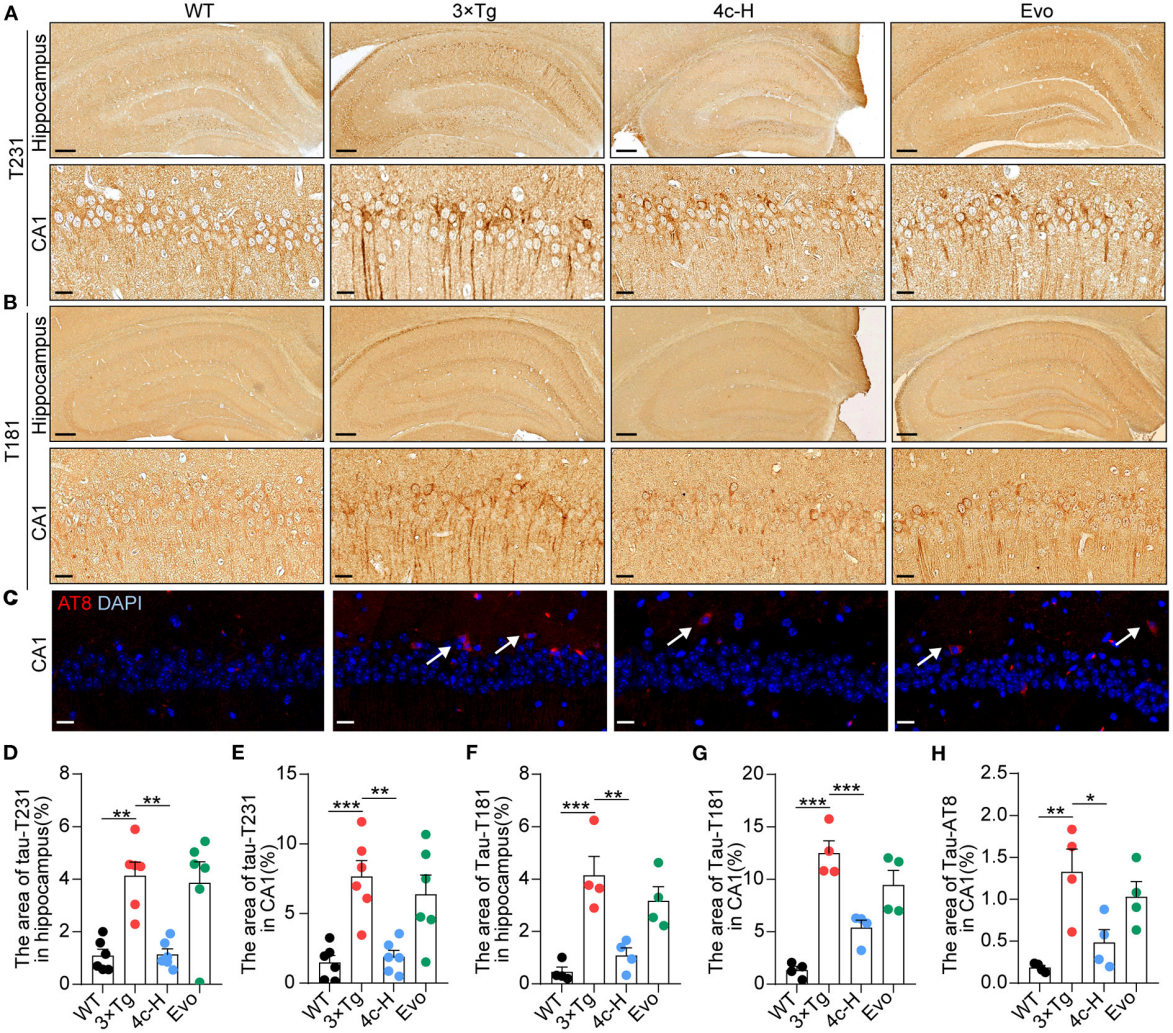
Determination of Tau phosphorylation in brain tissues. After the MWM test, mice from the WT, 3 x Tg, 4c-H, and Evo groups were sacrificed, and brain tissues were sampled for histological staining. **(A, B)** Tau phosphorylation was detected *via* immunohistochemical staining with T231 and T181 antibodies in the mouse hippocampus (scale bar = 200 μm) and hippocampal CA1 region (scale bar = 20 μm). **(C)** Tau phosphorylation was detected using immunofluorescent staining with the AT8 antibody in the mouse hippocampal CA1 region (white arrowheads indicate AT8-positive cells, scale bar = 20 μm). **(D–G)** Tau phosphorylation as detected using T231 and T181 antibodies in the hippocampus and the hippocampal CA1 region was quantified by the density of staining using ImageJ. **(H)** Tau phosphorylation as detected by the AT8 antibody was quantified by red fluorescence. Data are represented as mean ± SEM, *n* = 6; ^*^P < 0.05, ^**^P < 0.01, and ^***^P < 0.001.

In patients and mice with AD, Ser202/Thr205 (AT8), Thr231 (T231), and Thr181 (T181) of Tau are commonly phosphorylated sites (Hanger et al., 2007; Spillantini and Goedert, 2013; Fang et al., 2019). After the cognitive behavior analysis, the WT and 3 x Tg mice treated with vehicle, 200 μg/kg **4c**, or 200 μg/kg Evo were sacrificed, and Tau phosphorylation in hippocampal tissues was assessed by immunohistological staining. Compared with 3 x Tg mice treated with vehicle, compound **4c** reduced Tau hyperphosphorylation by 72.4 and 75.3% at T231 and 73.8 and 56.9% at T181 in the hippocampus and the hippocampal CA1 regions, respectively, and by 63.5% at AT8 in the hippocampal CA1 region in 3 x Tg mice (Figures 5A–H, *n* = 6; ^*^P < 0.05, ^**^P < 0.01, and ^***^P < 0.001). Compared with the 3 x Tg mice treated with vehicle, the 3 x Tg mice treated with Evo exhibited a mild reduction in Tau hyperphosphorylation (Figures 5D–H, *n* = 6).

### Compound 4c improves cognitive decline in APP/PS1 mice

The effects of **4c** and Evo on cognitive decline were assessed in APP/PS1 mice that express the APP^swe^ and PS1^Δ*E*9^ mutant genes and develop a more typical amyloid-dependent pathology than 3 × Tg AD mice (Billings et al., 2005; Tahara et al., 2006; Yuan et al., 2011; Huber et al., 2018; Fang et al., 2019). Seven-month-old APP/PS1 mice were treated with 200 μg/kg **4c** or Evo or vehicle for 4 weeks (Figure 4I). After treatment, *Y* maze and MWM tests were performed. In the *Y* maze test, **4c** treatment increased spontaneous alternations by 29.0% compared with the vehicle group (Figure 4J, *n* = 8; ^**^P < 0.01). In the MWM test, **4c** treatment reduced the time to look for the hidden platform, increased the number of platform crossings by 322.2%, and increased the target quadrant duration by 50.3% compared with the vehicle group (Figures 4K–M, *n* = 8; ^*^P < 0.05 and ^**^P < 0.01). In contrast, Evo did not improve the cognitive behavior of APP/PS1 mice in the *Y*-maze and MWM tests (Figures 4J–M, *n* = 8). Compared with WT mice, neither **4c** nor Evo treatment altered the swimming speed and distance of APP/PS1 mice (Figures 4N–P).

### Compound 4c reduces amyloid accumulation and glial cell aggregation in APP/PS1 mice

**Figure 6:**
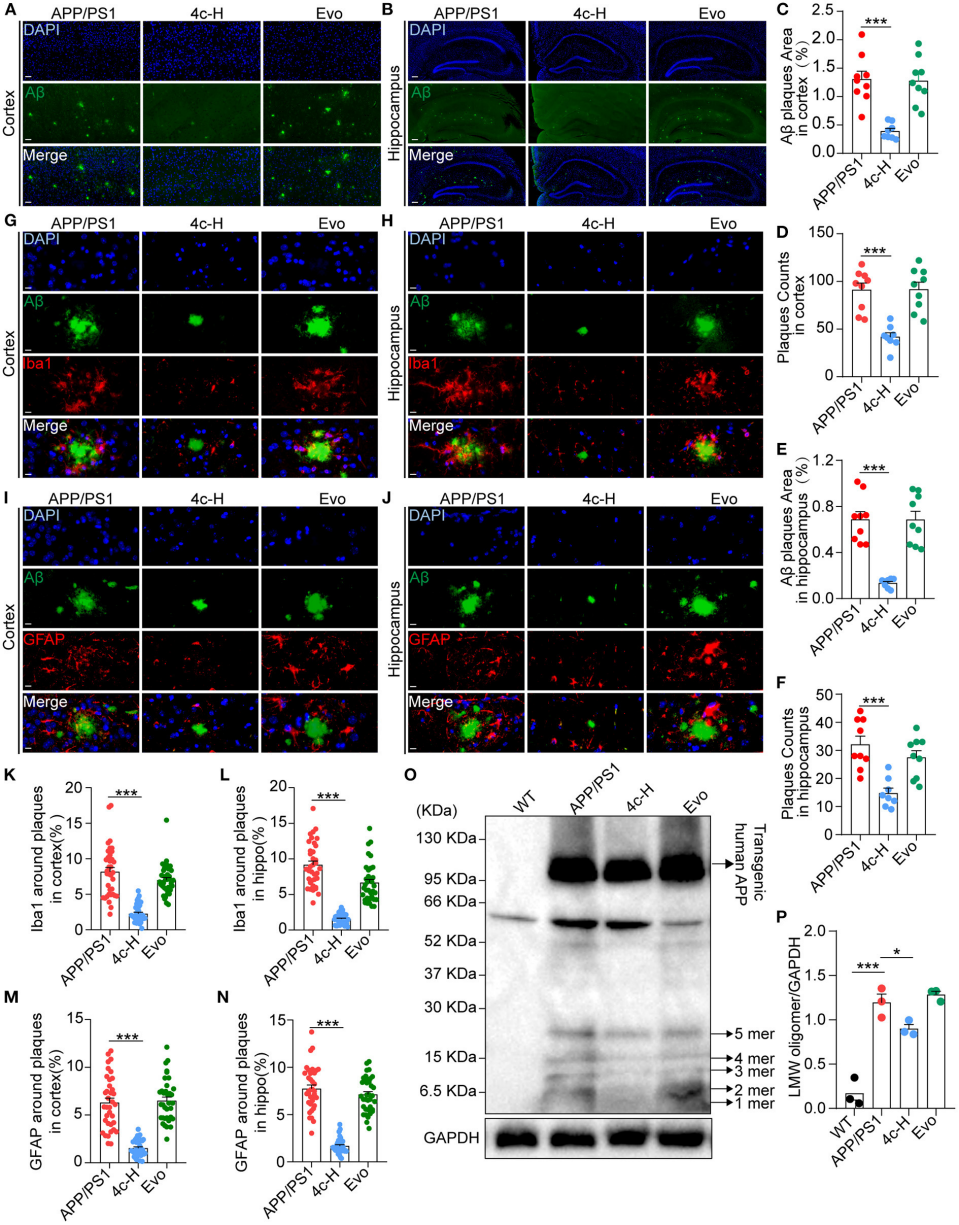
Observation of Aβ pathology in the brain in APP/PS1 mice. After the cognitive behavior evaluations, mice from the APP/PS1, 4c-H, and Evo groups were selectively sacrificed, and the paraffin sections of the brain were prepared by a standard pathological procedure. **(A, B)** Aβ in the cortex and hippocampus was observed by immunofluorescence staining with an anti-6E10 antibody (green) and counterstained with DAPI (scale bar = 200 μm). **(C–F)** The Aβ plaque area and plaque counts in the cortical and hippocampal regions were quantified by ImageJ (*n* = 9). **(G, H, K, L)** Microglia around Aβ plaques were observed by double immunofluorescence staining with anti-6E10 (green) and anti-Iba1 (red) antibodies in the cortex and hippocampus. The cell number of microglia around Aβ plaques was also counted (scale bar = 10 μm, *n* = 6). **(I, J, M, N)** The astrocytes around Aβ plaques were observed by double immunofluorescence staining with anti-6E10 (green) and anti-GFAP (red) antibodies in the cortex and the hippocampus, and the number of astrocytes around Aβ plaques was counted (scale bar = 10 μm, *n* = 6). **(O, P)** Low-molecular weight oligomers (LMW; 1–5 mers) of Aβ in brain tissues were detected by western blotting and quantified using ImageJ (*n* = 3). Data are represented as mean ± SEM; ^*^P < 0.05 and ^***^P < 0.001.

After the *Y*-maze and MWM tests, amyloid accumulation and glial cell aggregation were assessed in the cortex and the hippocampus of the APP/PS1 mice treated with vehicle, **4c**, or Evo. Immunofluorescent staining with an anti-6E10 antibody showed that, compared with the vehicle, **4c** treatment significantly reduced the Aβ plaque number by 54.1 and 54.1% and the Aβ area by 80.4% and 70.0% in the hippocampus and the cortex, respectively (Figures 6A–F, *n* = 6; ^***^P < 0.001). Furthermore, western blotting with an anti-6E10 antibody indicated that **4c** treatment significantly reduced the accumulation of AβOs in the brain by 24.8% compared with the vehicle (Figures 6O, P, *n* = 3; ^*^P < 0.05 and ^***^P < 0.001).

Amyloid β (Aβ) deposition in the brain is accompanied by gliosis in patients and mice with AD (Münch et al., 2003; Frost and Li, 2017), and Aβ-dependent gliosis and inflammation reflect the degree of injury in AD (Kim et al., 2015). Microglia and astrocytes in the brains of APP/PS1 mice were detected by immunofluorescent staining with anti-6E10, anti-Iba1, and anti-GFAP antibodies. Compared with the vehicle, **4c** significantly reduced microglial and astrocyte aggregation around Aβ plaques in the hippocampus and the cortex (Figures 6G–N, *n* = 6; ^***^P < 0.001). In contrast, Evo did not reduce amyloid accumulation or glial aggregation compared with the vehicle (Figures 6A–N, *n* = 6).

### Compound 4c modulates the expression of AD-related genes in 3 x Tg mice

**Figure 7:**
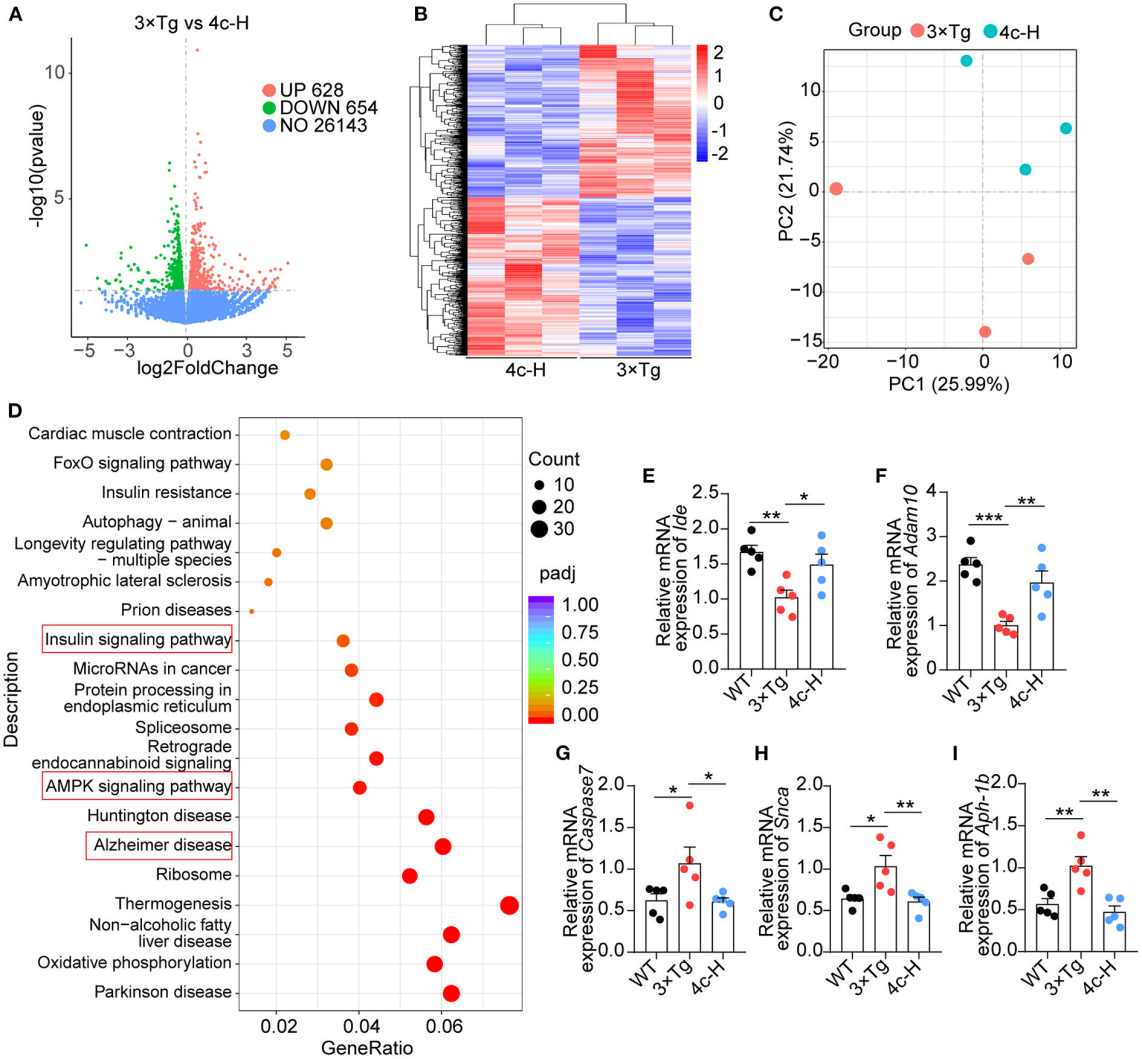
Transcriptome analysis of hippocampal tissues. Hippocampal tissues from 3 x Tg AD mice treated without or with **4c** (i.e., 3 x Tg and 4c-H groups) were selected for transcriptome analysis. **(A)** Volcano plots of DEGs between the 3 x Tg and 4c-H groups. DEGs were identified by comparing 3 x Tg and 4c-H mice using fold change >1 and *P*-value <0.05 as the cut-off (*n* = 3). **(B, C)** Hierarchical clustering and PCA analysis of the 3 x Tg and 4c-H groups were performed using the normalized RNA-seq read counts. **(D)** KEGG pathways enriched in the 628 and 654 genes that were upregulated and downregulated, respectively, in 4c-H mice compared with the 3 x Tg mice (*n* = 3). **(E–I)** AD-related genes (*Ide, Adam10, Caspase7, Snca*, and *Aph-1b*) were detected by RT-qPCR and compared among the WT, 3 x Tg, and 4c-H groups (*n* = 5). Data are represented as mean ± SEM; ^*^P < 0.05, ^**^P < 0.01, and ^***^P < 0.001.

The effects of **4c** on gene expression were investigated by performing an RNA sequencing (RNA-seq) analysis of the hippocampal tissues of three mice randomly selected from the 200 μg/kg **4c** treatment group and the vehicle group. The two groups were compared to identify differentially expressed genes (DEGs), and 628 upregulated and 654 downregulated DEGs were identified in the **4c** group (Figure 7A). The results of clustering and principal component analysis (PCA) showed that the **4c** group and the vehicle group were distinct (Figures 7B, C). KEGG analysis showed that the DEGs were enriched in genes related to AD, insulin signaling, AMPK signaling, and oxidative phosphorylation (Figure 7D). The AD-related genes identified by RNA-seq were further confirmed by RT-qPCR, which verified the downregulation of *Ide* and *Adam10* expression and the upregulation of *Caspase7, Snca*, and *Aph-1b* expression in 3 x Tg mice compared with WT mice. Treatment of 3 x Tg mice with **4c** completely recovered these changes in gene expression (Figures 7E–I, *n* = 5; ^*^P < 0.05, ^**^P < 0.01, and ^***^P < 0.001).

### Compound 4c compensates the dysfunction of PI3K/AKT/GSK3β signaling in 3 x Tg mice

**Figure 8:**
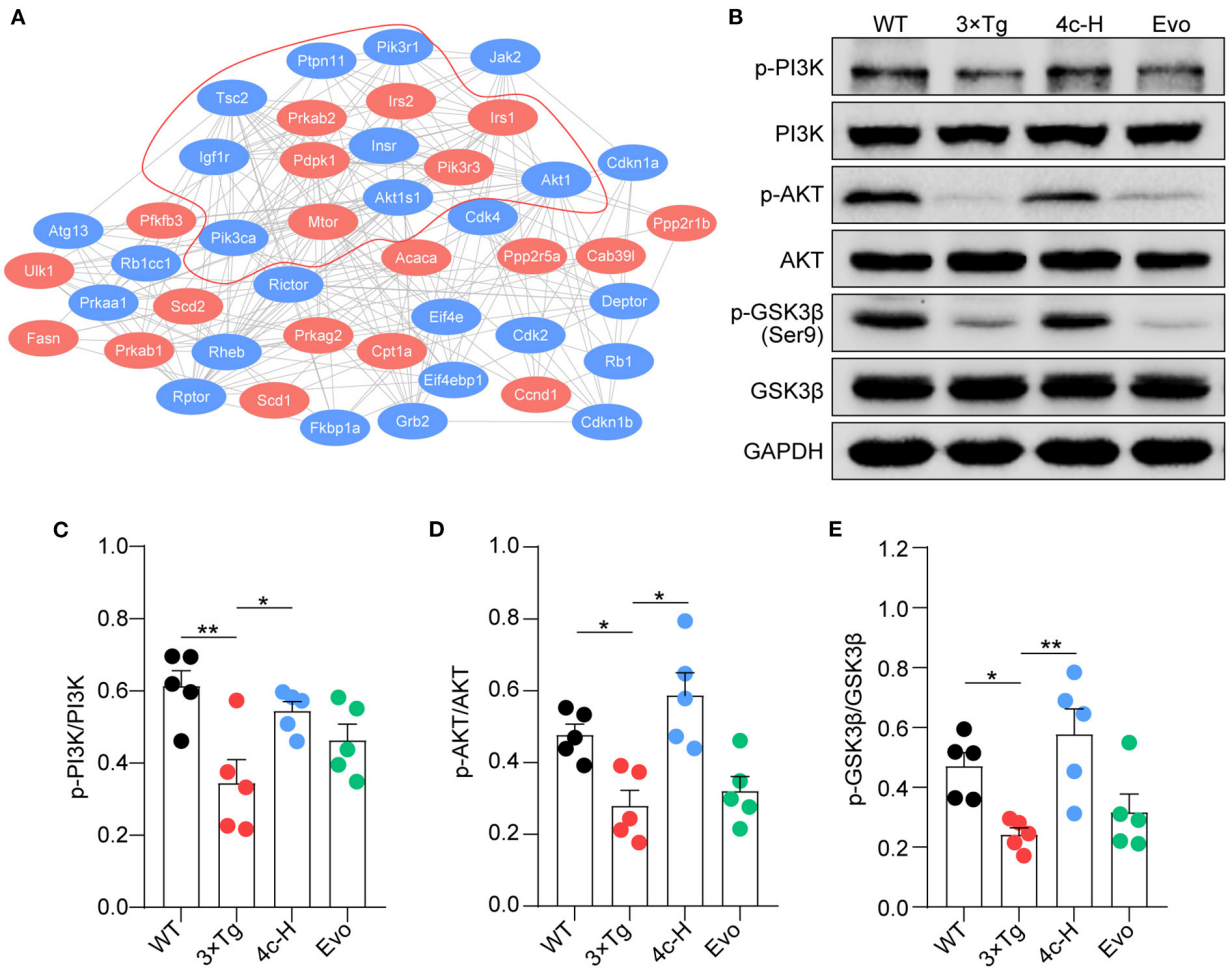
Compound **4c** activates the PI3K/AKT/GSK3β signaling pathway. **(A)** Protein–protein interaction network involving the AMPK signaling pathway. Compound **4c** affected the 19 proteins shown in red, and 25 interacting proteins identified by the STRING search are shown in blue. **(B)** Effects of **4c** treatment on p-PI3K, p-AKT, and p-GSK-3β protein expression in 3 x Tg mice. **(C–E)** Relative protein abundance of p-PI3K/PI3K, p-AKT/AKT, and p-GSK3β/GSK3β in each group. Data are the mean ± SEM, *n* = 5; ^*^P < 0.05 and ^**^P < 0.01 as indicated in the figure.

The insulin signaling pathway genes that were enriched in the KEGG analysis were used as seed genes for protein–protein interaction (PPI) network analysis. The PPI network constructed using STRING included 19 seed proteins and 25 interacting proteins in the insulin signaling pathway (Figure 8A) and suggested that compound **4c** may modulate the PI3K/AKT/GSK3β signaling pathway. Confirming the involvement of the PI3K/AKT/GSK3β signaling pathway, we found that PI3K and AKT phosphorylation were inhibited and that GSK3β was activated in 3 × Tg mice treated with vehicle compared with WT mice (Figures 8B–E, *n* = 5; ^*^P < 0.05 and ^**^P < 0.01). Compared with the vehicle treatment, **4c** treatment increased PI3K and AKT phosphorylation and inhibited GSK3β activation in 3 x Tg mice (Figures 8B–E, *n* = 5; ^*^P < 0.05 and ^**^P < 0.01).

### Compound 4c recovers PI3K/AKT/GSK3β/Tau dysfunction caused by Aβ *in vitro*

**Figure 9:**
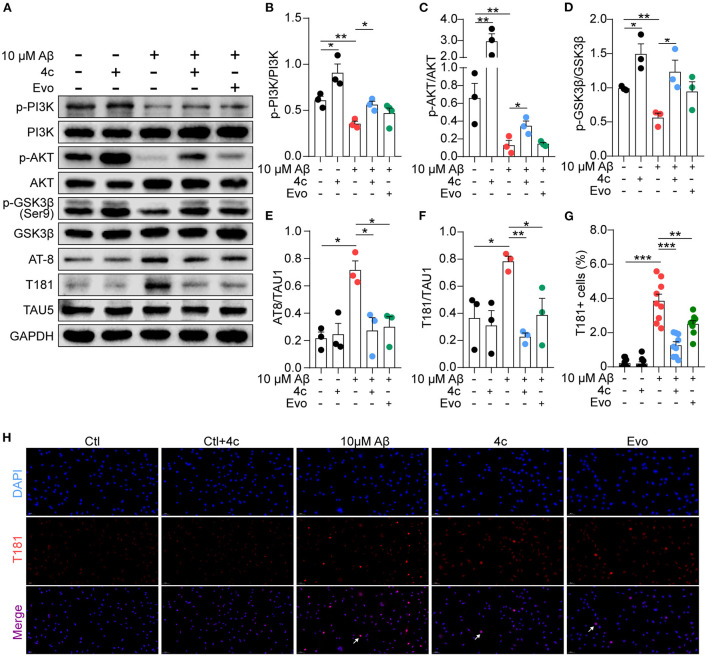
Determination of PI3K/AKT/GSK3β and Tau phosphorylation *in vitro*. A Tau phosphorylation cell model was established by treating SH-SY5Y cells with 10 μM Aβ1-42, and the cells were treated without or with **4c** (0.1 μg/ml) or Evo (0.1 μg/ml) for 24 h. **(A)** The phosphorylation of p-PI3K, p-AKT, p-GSK-3β, and Tau in SH-SY5Y cells was detected by western blot using antibodies recognizing AT-8, T181, and Tau5 (*n* = 3). **(B–F)** The phosphorylation ratios compared with total protein were estimated by ImageJ (*n* = 3). **(G, H)** Tau phosphorylation was detected by immunofluorescent staining with anti-T181 antibody (*n* = 9 fields). Data are represented as mean ± SEM; ^*^P < 0.05, ^**^P < 0.01, and ^***^P < 0.001.

Aβ stimulation induces PI3K/AKT/GSK3β dysfunction and results in Tau phosphorylation by regulating the PI3K/AKT/GSK3β pathway (Jeon et al., 2015; Zhao et al., 2019). Consequently, the effect of **4c** on this signaling pathway was examined in Aβ-treated SH-SY5Y cells. Aβ treatment reduced PI3K and AKT phosphorylation and increased GSK3β activation in SH-SY5Y cells (Figures 9A–D, *n* = 3; ^*^P < 0.05 and ^**^P < 0.01), resulting in Tau phosphorylation at AT-8 and T181 (Figures 9A, E, F, *n* = 3; ^*^P < 0.05 and ^**^P < 0.01). Treatment with **4c** dramatically reversed PI3K/AKT/GSK3β dysfunction and Tau phosphorylation induced by Aβ (Figures 9A–F, *n* = 3; ^*^P < 0.05 and ^**^P < 0.01, ^***^P < 0.001). The effect of Evo on Tau phosphorylation was smaller than that of **4c**. The ameliorative effect of **4c** on Tau phosphorylation was further confirmed by an immunofluorescence assay (Figures 9G, H, *n* = 3; ^***^P < 0.001).

## Discussion

We previously demonstrated that Evo improves AD phenotypes in a mouse model (Yuan et al., 2011). However, Evo exhibits side effects such as a reduction in hepatic cell apoptosis and impairment of the cardiovascular system (Yang et al., 2017). To reduce this cytotoxicity, we synthesized several evodiamine derivatives, compounds **3a−3k** and **4a−4j**, *via* heterocyclic substitution using 1,2-dimethoxybenzene to substitute the indole ring. The introduction of amide groups while maintaining the oxygen atom was also investigated. Structurally, compounds **3a−3k** and **4a−4j** could be considered bioisosteres. Supporting our design strategy, the amide compounds containing the amine group (**3i** and **4c**–**i**) displayed higher activities against H_2_O_2_ and AβOs *in vitro* with the exception of the double-substituted compound **4j**. Compounds **3i**, **4c**, and **4c** displayed lower toxicity than Evo. In particular, the LD_50_ of **4c** in SHSY-5Y and HepaG2 cells was three-fold higher than that of Evo (Figure 2). Compound **4c** also exhibited a lower effective dosage for reducing H_2_O_2_ cytotoxicity (Figure 2).

Based on these results, **4c** was further evaluated in 3 x Tg mice and APP/PS1 mice, which present typical Tau pathology and amyloid-dependent pathology, respectively (Billings et al., 2005; Tahara et al., 2006; Yuan et al., 2011; Huber et al., 2018; Fang et al., 2019). Treatment with **4c** significantly improved cognitive behavior disorder in both 3 x Tg mice and APP/PS1 mice, and the effective dose of **4c** in APP/PS1 mice was 500-fold lower than that of Evo described previously (Yuan et al., 2011) (Figure 4). In addition, treatment of 3 x Tg mice with **4c** significantly reduced the hyperphosphorylation of Tau at Ser202/Thr205, Thr231, and Thr181, the common sites of hyperphosphorylation in AD patients and AD mice (Hanger et al., 2007; Spillantini and Goedert, 2013; Fang et al., 2019) (Figure 5). In APP/PS1 mice, **4c** treatment significantly reduced the number of senile plaques and amyloid accumulation in the brain and glial aggregation around the plaques (Figure 6), indicating improvements in Aβ-dependent gliosis and neuroinflammation (Kim et al., 2015).

Furthermore, the RNA-seq analysis showed that genes related to AD, insulin signaling, AMPK signaling, and oxidative phosphorylation were enriched in the hippocampal tissues of 3 x Tg mice treated with **4c** (Figure 7). RT-qPCR confirmed that the changes in the expression of AD-related genes, including *Ide, Adam10, Caspase7, Snca*, and *Aph-1b*, in 3 x Tg mice were recovered by **4c** treatment (Figure 7).

KEGG analysis of the RNA sequencing data indicated that the insulin signaling pathway play a role in the mechanism of action of **4c**. The PI3K/AKT signaling pathway is an insulin signaling pathway that regulates glucose metabolism (Caruso et al., 2014). There is growing evidence that AD can be considered “type 3 diabetes” and that abnormal glucose metabolism is an important molecular event in the AD disease process (de la Monte and Wands, 2008; Sedzikowska and Szablewski, 2021). Interruption of the PI3K/AKT/GSK3β signaling pathway is a common event in the AD brain (de la Monte and Wands, 2008; Sedzikowska and Szablewski, 2021). The interruption of PI3K/AKT in AD may inhibit the mammalian target of rapamycin (mTOR), which is indirectly involved in amyloid accumulation (O' Neill, 2013; Cao et al., 2021; Querfurth and Lee, 2021). GSK3β is a typical downstream mediator that is inhibited by the PI3K/AKT pathway (Kitagishi et al., 2014). AKT dysfunction may cause abnormal GSK3β activation and Tau hyperphosphorylation in the brain of patients with AD (Takashima, 2006; Hanger et al., 2009; Querfurth and Lee, 2021). Overall, previous studies suggests that PI3K/AKT/GSK3β is a central signaling pathway in AD pathogenesis, namely, Aβ deposition and Tau hyperphosphorylation.

**Figure 10:**
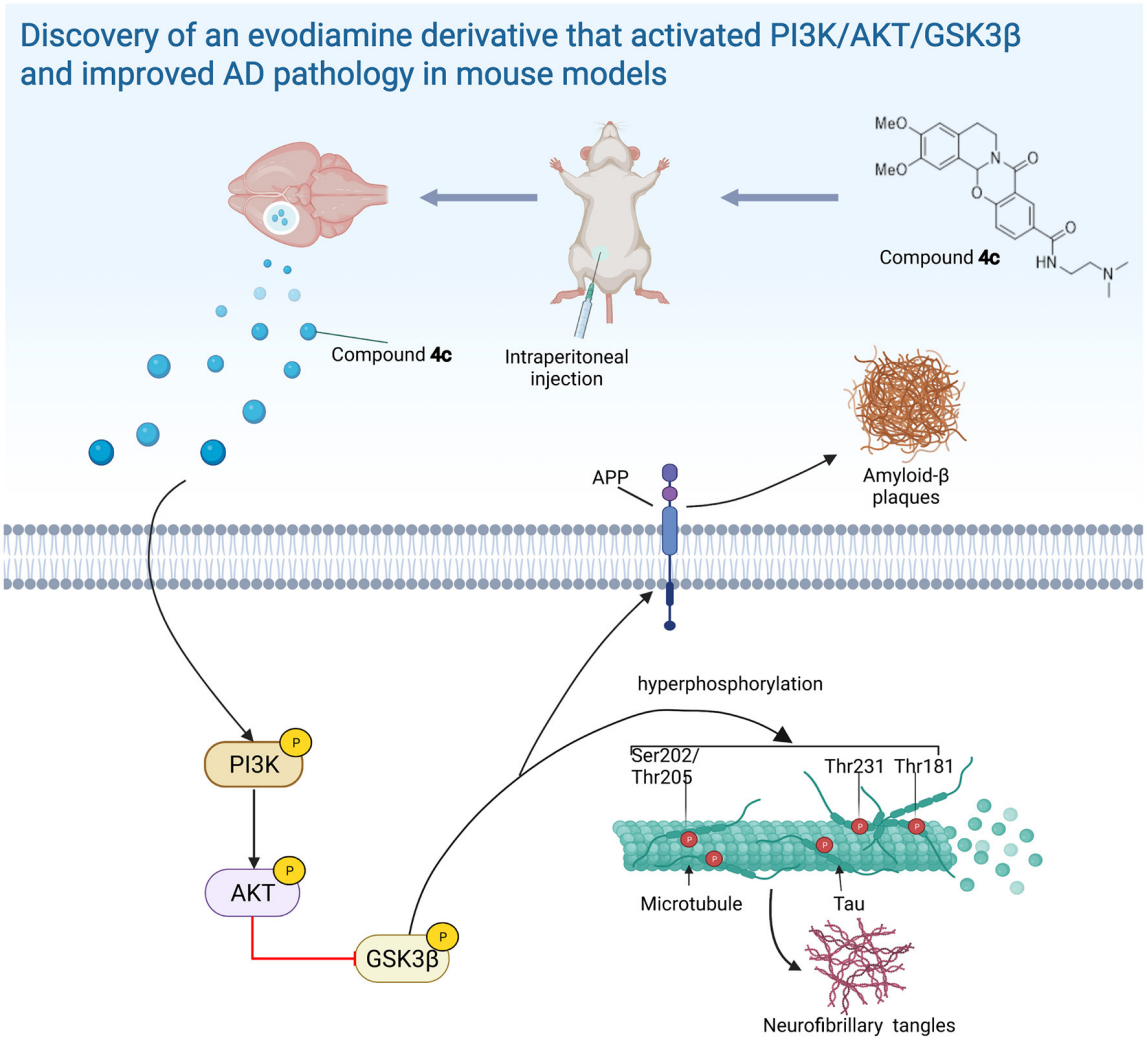
The evodiamine derivative **4c** improves AD pathology *via* the PI3K/AKT/GSK3β pathway. Compound **4c** reversed the impairment of the PI3K/AKT/GSK3β signaling pathway in AD mice, thereby improving AD pathology (diagram was created with BioRender.com).

We found that **4c** treatment recovered PI3K/AKT/GSK3β dysfunction in 3 x Tg mice (Figure 8). In SH-SY5Y cells, Aβ treatment reduced PI3K/AKT/GSK3β phosphorylation and induced Tau hyperphosphorylation, and **4c** treatment ameliorated these effects of Aβ treatment (Figure 9). Our *in vitro* and *in vivo* results suggest that the recovery of PI3K/AKT/GSK3β dysfunction is an important mechanism by which 4c mitigates the pathological features of AD (Figure 10).

## Conclusion

We successfully synthesized an Evo derivative, compound **4c**, *via* heterocyclic substitution and amide introduction that had much lower cytotoxicity and higher activity. Treatment with compound **4c** significantly improved the pathological features of AD in 3 x Tg mice and APP/PS1 mice and activated the PI3K/AKT/GSK3β pathway, a central signaling pathway related to AD pathogenesis. Overall, our results indicate that **4c** is a prospective compound for AD therapy based on the recovery of the PI3K/AKT/GSK3β pathway dysfunction.

## Data availability statement

The data presented in the study are deposited in the NCBI Sequence Read Archive (https://www.ncbi.nlm.nih.gov/sra, accession numbers SRR21384922, SRR21384923, SRR21384924, SRR21384925, SRR21384926, and SRR21384927).

## Ethics statement

The animal study was reviewed and approved by the Animal Care and Use Committee at the Institute of Laboratory Animal Science, Peking Union Medical College.

## Author contributions

LiaZ, YY, and SPang designed the research plan. YY, SL, and HC prepared the compounds. SPang, WD, SG, NL, XG, SPan, and XZ prepared the AD mice models. SPang and ZL performed mice treatment and behavior tests. SPang, XQ, and LiZ performed tissue collection, histology analyses, and mechanism exploration. LiaZ and YY audited the experimental procedures. LiaZ, YY, and SPang analyzed the data, wrote and/or revised the manuscript, and had primary responsibility for the final content. All authors contributed to the article and approved the submitted version.

## References

[B1] Bertram L. Tanzi R. E. (2020). Genomic mechanisms in Alzheimer's disease. Brain Pathol. 30, 966–977. 10.1111/bpa.12882 32657454 PMC8018017

[B2] Billings L. M. Oddo S. Green K. N. McGaugh J. L. LaFerla F. M. (2005). Intraneuronal abeta causes the onset of early Alzheimer's disease-related cognitive deficits in transgenic mice. Neuron 45, 675–688. 10.1016/j.neuron.2005.01.040 15748844

[B3] Cacace R. Sleegers K. Van Broeckhoven C. (2016). Molecular genetics of early-onset Alzheimer's disease revisited. Alzheimers Dement. 12, 733–48. 10.1016/j.jalz.2016.01.012 27016693

[B4] Cao B. Zeng M. Zhang Q. Zhang B. Cao Y. Wu Y.. (2021). Amentoflavone ameliorates memory deficits and abnormal autophagy in aβ(25-35)-induced mice by mtor signaling. Neurochem. Res. 46, 921–934. 10.1007/s11064-020-03223-8 33492604

[B5] Caruso M. Ma D. Msallaty Z. Lewis M. Seyoum B. Al-janabi W.. (2014). Increased interaction with insulin receptor substrate 1, a novel abnormality in insulin resistance and type 2 diabetes. Diabetes 63, 1933–1947. 10.2337/db13-1872 24584551 PMC4030113

[B6] Chiba-Falek O. Gottschalk W. K. Lutz M. W. (2018). The effects of the tomm40 poly-t alleles on Alzheimer's disease phenotypes. Alzheimers Dement. 14, 692–698. 10.1016/j.jalz.2018.01.015 29524426 PMC5938113

[B7] Chou C. H. Yang C. R. (2021). Neuroprotective studies of evodiamine in an okadaic acid-induced neurotoxicity. Int. J. Mol. Sci. 22, 5347. 10.3390/ijms22105347 34069531 PMC8161163

[B8] de la Monte S.M. Wands J.R. (2008). Alzheimer's disease is type 3 diabetes-evidence reviewe d. J. Diabetes Sci. Technol. 2, 1101–13. 10.1177/193229680800200619 19885299 PMC2769828

[B9] De Roeck A. Van Broeckhoven C. Sleegers K. (2019). The role of abca7 in Alzheimer's disease: evidence from genomics, transcriptomics and methylomics. Acta Neuropathol. 138, 201–220. 10.1007/s00401-019-01994-1 30903345 PMC6660495

[B10] Fang E. F. Hou Y. Palikaras K. Adriaanse B. A. Kerr J. S. Yang B.. (2019). Mitophagy inhibits amyloid-β and tau pathology and reverses cognitive deficits in models of Alzheimer's disease. Nat. Neurosci. 22, 401–412. 10.1038/s41593-018-0332-9 30742114 PMC6693625

[B11] Fang Z. Tang Y. Ying J. Tang C. Wang Q. (2020). Traditional chinese medicine for anti-Alzheimer's disease: berberine and evodiamine from evodia rutaecarpa. Chin. Med. 15, 82. 10.1186/s13020-020-00359-1 32774447 PMC7409421

[B12] Frost G. R. Li Y. M. (2017). The role of astrocytes in amyloid production and Alzheimer's disease. Open Biol. 7, 170228. 10.1098/rsob.170228 29237809 PMC5746550

[B13] Gavaraskar K. Dhulap S. Hirwani R. R. (2015). Therapeutic and cosmetic applications of evodiamine and its derivatives–a patent review. Fitoterapia 106, 22–35. 10.1016/j.fitote.2015.07.019 26255828

[B14] GBD 2016 Dementia Collaborators. (2019). Global, regional, and national burden of Alzheimer's disease and other dementias, 1990-2016: a systematic analysis for the global burden of disease study 2016. Lancet Neurol. 18, 88–106. 10.1016/S1474-4422(18)30403-4 30497964 PMC6291454

[B15] Hanger D. P. Anderton B. H. Noble W. (2009). Tau phosphorylation: the therapeutic challenge for neurodegenerative disease. Trends Mol. Med. 15, 112–9. 10.1016/j.molmed.2009.01.003 19246243

[B16] Hanger D. P. Byers H. L. Wray S. Leung K. Y. Saxton M. J. Seereeram A.. (2007). Novel phosphorylation sites in tau from Alzheimer brain support a role for casein kinase 1 in disease pathogenesis. J. Biol. Chem. 282, 23645–54. 10.1074/jbc.M703269200 17562708

[B17] Huber C. M. Yee C. May T. Dhanala A. Mitchell C. S. (2018). Cognitive decline in preclinical Alzheimer's disease: amyloid-beta vs. tauopathy. J. Alzheimers Dis. 61, 265–281. 10.3233/JAD-170490 29154274 PMC5734131

[B18] Jeon S. Park J. E. Lee J. Liu Q. F. Jeong H. J. Pak S. C.. (2015). Illite improves memory impairment and reduces aβ level in the tg-appswe/ps1de9 mouse model of Alzheimer?s disease through akt/creb and gsk-3β phosphorylation in the brain. J. Ethnopharmacol. 160, 69–77. 10.1016/j.jep.2014.11.029 25457987

[B19] Kim H. Y. Kim H. V. Jo S. Lee C. J. Choi S. Y. Kim D. J.. (2015). Epps rescues hippocampus-dependent cognitive deficits in app/ps1mice by disaggregation of amyloid-β oligomers and plaques. Nat. Commun. 6, 8997. 10.1038/ncomms9997 26646366 PMC4686862

[B20] Kim J. H. (2018). Genetics of Alzheimer's disease. Dement. Neurocogn. Disord. 17, 131–136. 10.12779/dnd.2018.17.4.131 30906402 PMC6425887

[B21] Kitagishi Y. Nakanishi A. Ogura Y. Matsuda S. (2014). Dietary regulation of pi3k/akt/gsk-3β pathway in Alzheimer's disease. Alzheimers Res. Ther. 6, 35. 10.1186/alzrt265 25031641 PMC4075129

[B22] Lu D. Wang J. Li J. Guan F. Zhang X. Dong W.. (2018). Meox1 accelerates myocardial hypertrophic decompensation through gata4. Cardiovasc. Res. 114, 300–311. 10.1093/cvr/cvx222 29155983

[B23] Luo R. Su L. Y. Li G. Yang J. Liu Q. Yang L. X.. (2020). Activation of ppara-mediated autophagy reduces Alzheimer disease-like pathology and cognitive decline in a murine model. Autophagy 16, 52–69. 10.1080/15548627.2019.1596488 30898012 PMC6984507

[B24] Münch G. Apelt J. Rosemarie Kientsch E. Stahl P. Lüth H. J. Schliebs R.. (2003). Advanced glycation endproducts and pro-inflammatory cytokines in transgenic tg2576 mice with amyloid plaque pathology. J. Neurochem. 86, 283–9. 10.1046/j.1471-4159.2003.01837.x 12871569

[B25] Nie Y. Luo D. Yang M. Wang Y. Xiong L. Gao L.. (2017). A meta-analysis on the relationship of the pon genes and Alzheimer disease. J. Geriatr. Psychiatry Neurol. 30, 303–310. 10.1177/0891988717731825 28954597

[B26] O' Neill C. (2013). Pi3-kinase/akt/mtor signaling: impaired on/off switches in aging, cognitive decline and Alzheimer's disease. Exp. Gerontol. 48, 647–653. 10.1016/j.exger.2013.02.025 23470275

[B27] Pang S. Dong W. Liu N. Gao S. Li J. Zhang X.. (2021). Diallyl sulfide protects against dilated cardiomyopathy *via* inhibition of oxidative stress and apoptosis in mice. Mol. Med. Rep. 24, 852. 10.3892/mmr.2021.12492 34651661 PMC8532119

[B28] Pang S. Sun C. Gao S. Yang Y. Pan X. Zhang L.. (2020). Evodiamine derivatives improve cognitive abilities in app(swe)/ps1(δe9) transgenic mouse models of Alzheimer's disease. Animal Model Exp. Med. 3, 193–199. 10.1002/ame2.12126 32613178 PMC7323704

[B29] Querfurth H. Lee H. K. (2021). Mammalian/mechanistic target of rapamycin (mtor) complexes in neurodegeneration. Mol. Neurodegener. 16, 44. 10.1186/s13024-021-00428-5 34215308 PMC8252260

[B30] Rodriguez-Garcia A. Lynn R. C. Poussin M. Eiva M. A. Shaw L. C. O'Connor R. S.. (2021). Car-t cell-mediated depletion of immunosuppressive tumor-associated macrophages promotes endogenous antitumor immunity and augments adoptive immunotherapy. Nat. Commun. 12, 877. 10.1038/s41467-021-20893-2 33563975 PMC7873057

[B31] Sedzikowska A. Szablewski L. (2021). Insulin and insulin resistance in Alzheimer's disease. Int. J. Mol. Sci. 22, 9987. 10.3390/ijms22189987 34576151 PMC8472298

[B32] Silva M. V. F. Loures C. M. G. Alves L. C. V. de Souza L. C. Borges K. B. G. Carvalho M. D. G. (2019). Alzheimer's disease: risk factors and potentially protective measures. J. Biomed. Sci. 26, 33. 10.1186/s12929-019-0524-y 31072403 PMC6507104

[B33] Spangenberg E. Severson P. L. Hohsfield L. A. Crapser J. Zhang J. Burton E. A.. (2019). Sustained microglial depletion with csf1rinhibitor impairs parenchymal plaque development in an Alzheimer's disease model. Nat. Commun. 10, 3758. 10.1038/s41467-019-11674-z 31434879 PMC6704256

[B34] Spillantini M. G. Goedert M. (2013). Tau pathology and neurodegeneration. Lancet Neurol. 12, 609–22. 10.1016/S1474-4422(13)70090-5 23684085

[B35] Tahara K. Kim H. D. Jin J. J. Maxwell J. A. Li L. Fukuchi K.. (2006). Role of toll-like receptor signalling in abeta uptake and clearance. Brain 129, 3006–3019. 10.1093/brain/awl249 16984903 PMC2445613

[B36] Takashima A. (2006). Gsk-3 is essential in the pathogenesis of Alzheimer's disease. J. Alzheimers Dis. 9, 309–17. 10.3233/JAD-2006-9S335 16914869

[B37] Wang Z. Y. Liu J. G. Li H. Yang H. M. (2016). Pharmacological effects of active components of chinese herbal medicine in the treatment of Alzheimer's disease: a review. Am. J. Chin. Med. 44, 1525–1541. 10.1142/S0192415X16500853 27848250

[B38] Yang W. Ma L. Li S. Cui K. Lei L. Ye Z.. (2017). Evaluation of the cardiotoxicity of evodiamine *in vitro* and *in vivo*. Molecules 22, 943. 10.3390/molecules22060943 28598372 PMC6152646

[B39] Yang Y. Zhu C. Zhang M. Huang S. Lin J. Pan X.. (2016). Condensation of anthranilic acids with pyridines to furnish pyridoquinazolones *via* pyridine dearomatization. Chem. Commun. 52, 12869–12872. 10.1039/C6CC07365D 27735960

[B40] Yuan S. M. Gao K. Wang D. M. Quan X. Z. Liu J. N. Ma C. M.. (2011). Evodiamine improves congnitive abilities in samp8 and app(swe)/ps1(deltae9) transgenic mouse models of Alzheimer's disease. Acta Pharmacol. Sin. 32, 295–302. 10.1038/aps.2010.230 21278785 PMC4002780

[B41] Zhang L. Sun C. Jin Y. Gao K. Shi X. Qiu W.. (2017). Dickkopf 3 (dkk3) improves amyloid-β pathology, cognitive dysfunction, and cerebral glucose metabolism in a transgenic mouse model of Alzheimer's disease. J. Alzheimers Dis. 60, 733–746. 10.3233/JAD-161254 28922151

[B42] ZhangX. X.TianY.WangZ. T.MaY. H.TanL.YuJ. T.. (2021). The epidemiology of Alzheimer's disease modifiable risk factors and prevention. J. Prev. Alzheimers Dis. 8, 313–321. 10.14283/jpad.2021.1534101789

[B43] Zhang Y. Wang J. Wang C. Li Z. Liu X. Zhang J.. (2018). Pharmacological basis for the use of evodiamine in Alzheimer's disease: antioxidation and antiapoptosis. Int. J. Mol. Sci. 19, 1527. 10.3390/ijms19051527 29883380 PMC5983845

[B44] Zhao Z. Y. Zhang Y. Q. Zhang Y. H. Wei X. Z. Wang H. Zhang M.. (2019). The protective underlying mechanisms of schisandrin on sh-sy5y cell model of Alzheimer's disease. J. Toxicol. Environ. Health A 82, 1019–1026. 10.1080/15287394.2019.1684007 31739764

