# Supplementary material for: Discovery of an evodiamine derivative for PI3K/AKT/GSK3β pathway activation and AD pathology improvement in mouse models
Source: Front Mol Neurosci. 2023 Jan 9;15:1025066. doi: 10.3389/fnmol.2022.1025066 (PMC9868638; doi:10.3389/fnmol.2022.1025066)
Supplement: Supplementary file 1 [file Data_Sheet_1.docx]

**Supporting Information**

**Discovery of an evodiamine derivative activated PI3K/AKT/GSK3β and improved AD pathology in mouse model**

Shuo Pang^1, 4^, Siyuan Li^3^, Hanzeng Cheng^3^, Zhuohui Luo^1, 4^, Xiaolong Qi^1, 4^, Feifei Guan^1, 4^, Wei Dong^1^, Shan Gao^1^, Ning Liu^4^, Xiang Gao^4^, Shuo Pan^4^, Xu Zhang^4^, Li Zhang^1, 4^, Yajun Yang^3*^, Lianfeng Zhang^1, 2*^

^1^Key Laboratory of Human Disease Comparative Medicine, National Health Commission of China (NHC), Institute of Laboratory Animal Science, Peking Union Medical College, Chinese Academy of Medical Sciences, Beijing 100021, China.

^2^Neuroscience center, Chinese Academy of Medical Sciences, Beijing 100730, China.

^3^Beijing Key Laboratory of Active Substance Discovery and Drug ability Evaluation, Institute of Material Medical, Chinese Academy of Medical Sciences and Peking Union Medical College, Xiannongtan Street 1, Beijing 100050, China

^4^Beijing Engineering Research Center for Experimental Animal Models of Human Diseases, Institute of Laboratory Animal Science, Peking Union Medical College, Chinese Academy of Medical Sciences, Beijing 100021, China.

**^*^Corresponding author:**

Yajun Yang and Lianfeng Zhang.

Building 5, PanjiayuanNanli, Chaoyang District, Beijing 100021, China.

*E-mail*: [yangyajun@imm.ac.cn](mailto:yangyajun@imm.ac.cn) and [zhanglf@cnilas.org](mailto:zhanglf@cnilas.org); Phone: 86-010-87778442; Fax: 86-010-67776394.

**1. Chemistry**

- 1. *General procedure for the synthesis of compounds* ***3a****-****3k***

To a solution of 6,7-dimethoxy-3,4-dihydroisoquinoline (1.0 equiv) and 5-nitrosalicylic acid (1.1 equiv) in dichloromethane was added 1-(3-dimethylaminopropyl)-3-ethylcarbodiimide hydrochloride (2.0 equiv). The mixture was stirred at room temperature for 8 h. After completion of the reaction, the mixture was diluted with dichloromethane. Subsequently, the mixture was washed with a saturated solution of NaHCO_3_, H_2_O, and a saturated solution of NaCl. The dichloromethane phase was dried over anhydrous Na_2_SO_4_, filtered and concentrated under vacuum. The crude product was purified by silica gel column chromatography to obtain **6**, yield 85.5%. ^1^H NMR (400 MHz, DMSO-*d*_6_) δ 8.60 (d, *J* = 2.8 Hz, 1H), 8.42 (dd, *J* = 9.0, 2.9 Hz, 1H), 7.41 (d, *J* = 9.0 Hz, 1H), 7.14 (s, 1H), 6.92 (s, 1H), 6.58 (s, 1H), 4.42 (ddd, *J* = 12.8, 4.8, 3.0 Hz, 1H), 3.80 (d, *J* = 2.1 Hz, 6H), 3.25 (ddd, *J* = 12.8, 11.1, 4.0 Hz, 1H), 2.92 (ddd, *J* = 15.9, 11.1, 4.8 Hz, 1H), 2.86 – 2.77 (m, 1H).

To a solution of **6** (1.0 equiv) in MeOH was added 10% Pd/C (0.1 equiv). The mixture was stirred under hydrogen at room temperature for 12 h. After completion of the reaction, the mixture was filtered and evaporated under reduced pressure. The crude product was purified by silica gel column chromatography to obtain **7**, yield 78.5%. ^1^H NMR (400 MHz, DMSO-*d*_6_) δ 7.11 – 6.99 (m, 2H), 6.96 – 6.71 (m, 3H), 6.16 (s, 1H), 5.04 (s, 2H), 4.33 (dt, *J* = 13.0, 4.1 Hz, 1H), 3.19 (td, *J* = 13.0, 11.8, 3.9 Hz, 1H), 2.86 (ddd, *J* = 15.6, 10.7, 4.7 Hz, 1H), 2.76 (dt, *J* = 15.7, 3.7 Hz, 1H).

To a solution of **7** (1.0 equiv) with different acids, acyl chlorides, or isocyanates (1.1 equiv) in dichloromethane was added N,N-Diisopropylethylamine (3.0 equiv). For different acids, 2-(7-azabenzotriazol-1-yl)-N,N,N',N'-tetramethyluronium hexafluorophosphate (1.5 equiv) was added separately. The mixture was stirred at room temperature for 12 h. After completion of the reaction, the mixture was diluted with dichloromethane. Subsequently, the mixture was washed with a saturated solution of NaHCO_3_, H_2_O, and a saturated solution of NaCl. The dichloromethane phase was dried over anhydrous Na_2_SO_4_, filtered and concentrated under vacuum. The crude product was purified by silica gel column chromatography to obtain **3a**-**3k.**

*N-(2,3-dimethoxy-8-oxo-5,13a-dihydro-6H,8H-benzo[5,6][1,3]oxazino[2,3-a]isoquinolin-10-yl)benzamide (****3a****)* ^1^H NMR (400 MHz, DMSO-*d*_6_) δ 10.37 (s, 1H), 8.32 (d, *J* = 2.6 Hz, 1H), 8.06 – 7.92 (m, 3H), 7.66 – 7.49 (m, 3H), 7.17 (d, *J* = 8.8 Hz, 1H), 7.11 (s, 1H), 6.91 (s, 1H), 6.36 (s, 1H), 4.43 – 4.35 (m, 1H), 3.80 (s, 6H), 3.29 – 3.18 (m, 1H), 2.96 – 2.85 (m, 1H), 2.85 – 2.75 (m, 1H). ^13^C NMR (100 MHz, DMSO-*d*_6_) δ 165.93, 162.49, 153.70, 149.86, 148.18, 135.06, 134.44, 132.16, 129.25, 128.92, 128.09, 127.13, 122.67, 119.86, 118.72, 117.31, 111.73, 111.60, 84.26, 56.22, 56.03, 38.58, 27.71. HR-MS (ESI) *m/z*: calcd for C_25_H_23_O_5_N_2_ [M+H]^+^, 431.1601; found, 431.1598.

*N-(2,3-dimethoxy-8-oxo-5,13a-dihydro-6H,8H-benzo[5,6][1,3]oxazino[2,3-a]isoquinolin-10-yl)benzofuran-2-carboxamide (****3b****)* ^1^H NMR (400 MHz, DMSO-*d*_6_) δ 10.68 (s, 1H), 8.35 (d, *J* = 2.6 Hz, 1H), 8.01 (dd, *J* = 8.9, 2.6 Hz, 1H), 7.88 – 7.69 (m, 3H), 7.52 (ddd, *J* = 8.4, 7.2, 1.3 Hz, 1H), 7.43 – 7.33 (m, 1H), 7.20 (d, *J* = 8.8 Hz, 1H), 7.11 (s, 1H), 6.91 (s, 1H), 6.37 (s, 1H), 4.39 (dt, *J* = 12.7, 4.0 Hz, 1H), 3.81 (s, 6H), 3.29 – 3.17 (m, 1H), 2.91 (ddd, *J* = 15.6, 10.8, 4.8 Hz, 1H), 2.85 – 2.75 (m, 1H). ^13^C NMR (100 MHz, DMSO-*d*_6_) δ 162.42, 157.14, 154.94, 153.99, 149.87, 149.02, 148.18, 133.58, 129.27, 127.73, 127.58, 127.25, 124.38, 123.44, 122.61, 120.05, 118.80, 117.48, 112.44, 111.74, 111.62, 111.22, 84.29, 56.23, 56.04, 40.45, 40.24, 40.04, 39.83, 39.62, 39.41, 39.20, 38.61, 27.71. HR-MS (ESI) *m/z*: calcd for C_27_H_23_O_6_N_2_ [M+H]^+^, 471.1551; found, 471.1552.

*N-(2,3-dimethoxy-8-oxo-5,13a-dihydro-6H,8H-benzo[5,6][1,3]oxazino[2,3-a]isoquinolin-10-yl)-1H-indole-2-carboxamide (****3c****)* ^1^H NMR (400 MHz, DMSO-*d*_6_) δ 11.74 (d, *J* = 2.3 Hz, 1H), 10.35 (s, 1H), 8.32 (d, *J* = 2.7 Hz, 1H), 8.04 (dd, *J* = 8.8, 2.7 Hz, 1H), 7.69 (d, *J* = 8.0 Hz, 1H), 7.54 – 7.35 (m, 2H), 7.29 – 7.15 (m, 2H), 7.15 – 7.00 (m, 2H), 6.91 (s, 1H), 6.37 (s, 1H), 4.40 (dt, *J* = 12.7, 4.3 Hz, 1H), 3.81 (s, 6H), 3.24 (ddd, *J* = 12.7, 10.9, 3.9 Hz, 1H), 2.92 (ddd, *J* = 15.7, 10.9, 4.8 Hz, 1H), 2.87 – 2.73 (m, 1H). ^13^C NMR (100 MHz, DMSO-*d*_6_) δ 162.48, 160.11, 153.57, 149.89, 148.21, 137.32, 134.34, 131.76, 129.27, 127.53, 126.69, 124.29, 122.71, 122.26, 120.41, 119.46, 118.84, 117.41, 112.88, 111.78, 111.66, 104.34, 84.29, 56.25, 56.06, 38.59, 27.75. HR-MS (ESI) *m/z*: calcd for C_27_H_24_O_5_N_3_ [M+H]^+^, 470.1710; found, 470.1713.

*N-(2,3-dimethoxy-8-oxo-5,13a-dihydro-6H,8H-benzo[5,6][1,3]oxazino[2,3-a]isoquinolin-10-yl)acrylamide (****3d****)* ^1^H NMR (400 MHz, DMSO-*d*_6_) δ 10.26 (s, 1H), 8.20 (d, *J* = 2.6 Hz, 1H), 7.85 (dd, *J* = 8.8, 2.7 Hz, 1H), 7.21 – 7.02 (m, 2H), 6.90 (s, 1H), 6.48 – 6.20 (m, 3H), 5.77 (dd, *J* = 10.0, 2.1 Hz, 1H), 4.37 (ddd, *J* = 12.8, 4.7, 3.3 Hz, 1H), 3.80 (d, *J* = 1.6 Hz, 6H), 3.22 (ddd, *J* = 12.8, 10.9, 4.0 Hz, 1H), 2.90 (ddd, *J* = 15.6, 10.9, 4.8 Hz, 1H), 2.83 – 2.73 (m, 1H). ^13^C NMR (100 MHz, DMSO-*d*_6_) δ 163.76, 162.47, 153.59, 149.82, 148.13, 134.20, 131.93, 129.25, 127.76, 126.16, 122.57, 118.77, 117.57, 111.68, 111.54, 84.22, 56.18, 56.01, 38.60, 27.64. HR-MS (ESI) *m/z*: calcd for C_21_H_21_O_5_N_2_ [M+H]^+^, 381.1445; found, 381.1438.

*N-(2,3-dimethoxy-8-oxo-5,13a-dihydro-6H,8H-benzo[5,6][1,3]oxazino[2,3-a]isoquinolin-10-yl)cinnamamide (****3e****)* ^1^H NMR (400 MHz, DMSO-*d*_6_) δ 10.35 (s, 1H), 8.24 (d, *J* = 2.6 Hz, 1H), 7.88 (dd, *J* = 8.8, 2.7 Hz, 1H), 7.67 – 7.56 (m, 3H), 7.50 – 7.40 (m, 3H), 7.16 (d, *J* = 8.8 Hz, 1H), 7.10 (s, 1H), 6.90 (s, 1H), 6.81 (d, *J* = 15.7 Hz, 1H), 6.34 (s, 1H), 4.38 (ddd, *J* = 12.9, 4.7, 3.3 Hz, 1H), 3.23 (ddd, *J* = 12.8, 11.0, 4.0 Hz, 1H), 2.95 – 2.84 (m, 1H), 2.84 – 2.75 (m, 1H). ^13^C NMR (100 MHz, DMSO-*d*_6_) δ 163.97, 162.44, 153.48, 149.86, 148.17, 140.82, 135.07, 134.57, 130.36, 129.52, 129.26, 128.25, 125.91, 122.65, 122.40, 118.86, 118.57, 117.57, 111.73, 111.60, 84.34, 56.22, 56.03, 38.58, 27.71. HR-MS (ESI) *m/z*: calcd for C_27_H_25_O_5_N_2_ [M+H]^+^, 457.1758; found, 457.1758.

*N-(2,3-dimethoxy-8-oxo-5,13a-dihydro-6H,8H-benzo[5,6][1,3]oxazino[2,3-a]isoquinolin-10-yl)acetamide (****3f****)* ^1^H NMR (400 MHz, DMSO-*d*_6_) δ 10.04 (s, 1H), 8.10 (d, *J* = 2.6 Hz, 1H), 7.74 (dd, *J* = 8.8, 2.6 Hz, 1H), 7.14 – 7.03 (m, 2H), 6.89 (s, 1H), 6.31 (s, 1H), 4.41 – 4.29 (m, 1H), 3.79 (d, *J* = 1.9 Hz, 6H), 3.21 (ddd, *J* = 12.7, 10.9, 3.9 Hz, 1H), 2.89 (ddd, *J* = 15.5, 10.9, 4.7 Hz, 1H), 2.78 (dt, *J* = 15.7, 3.7 Hz, 1H), 2.05 (s, 3H). ^13^C NMR (100 MHz, DMSO-*d*_6_) δ 168.79, 162.46, 153.19, 149.84, 148.16, 134.63, 129.23, 125.73, 122.68, 118.72, 118.32, 117.36, 111.72, 111.59, 84.19, 56.21, 56.03, 38.54, 27.70, 24.30. HR-MS (ESI) *m/z*: calcd for C_20_H_21_O_5_N_2_ [M+H]^+^, 369.1445; found, 369.1443.

*N-(2,3-dimethoxy-8-oxo-5,13a-dihydro-6H,8H-benzo[5,6][1,3]oxazino[2,3-a]isoquinolin-10-yl)-3-phenylpropanamide (****3g****)* ^1^H NMR (400 MHz, DMSO-*d*_6_) δ 10.04 (s, 1H), 8.11 (d, *J* = 2.6 Hz, 1H), 7.74 (dd, *J* = 8.9, 2.7 Hz, 1H), 7.36 – 7.22 (m, 4H), 7.19 (t, *J* = 7.0 Hz, 1H), 7.14 – 7.03 (m, 2H), 6.90 (s, 1H), 6.31 (s, 1H), 4.36 (dd, *J* = 11.0, 6.7 Hz, 1H), 3.21 (t, *J* = 11.6 Hz, 1H), 2.95 – 2.75 (m, 4H), 2.62 (t, *J* = 7.8 Hz, 2H). ^13^C NMR (100 MHz, DMSO-*d*_6_) δ 170.86, 162.46, 153.23, 149.83, 148.16, 141.55, 134.49, 129.23, 128.81, 128.73, 126.46, 125.83, 122.67, 118.72, 118.43, 117.38, 111.72, 111.57, 84.20, 56.21, 56.03, 38.55, 38.31, 31.27, 27.69. HR-MS (ESI) *m/z*: calcd for C_27_H_27_O_5_N_2_ [M+H]^+^, 459.1914; found, 459.1919.

*N-(2,3-dimethoxy-8-oxo-5,13a-dihydro-6H,8H-benzo[5,6][1,3]oxazino[2,3-a]isoquinolin-10-yl)-2-phenoxyacetamide (****3h****)* ^1^H NMR (400 MHz, DMSO-*d*_6_) δ 10.22 (s, 1H), 8.19 (d, *J* = 2.7 Hz, 1H), 7.83 (dd, *J* = 8.8, 2.6 Hz, 1H), 7.39 – 7.28 (m, 2H), 7.15 (d, *J* = 8.7 Hz, 1H), 7.09 (s, 1H), 7.06 – 6.94 (m, 3H), 6.90 (s, 1H), 6.34 (s, 1H), 4.70 (s, 2H), 3.80 (d, *J* = 1.5 Hz, 6H), 3.27 – 3.14 (m, 1H), 2.97 – 2.84 (m, 1H), 2.79 (dt, *J* = 15.7, 3.7 Hz, 1H). ^13^C NMR (100 MHz, DMSO-*d*_6_) δ 167.16, 162.41, 158.16, 153.73, 149.84, 148.15, 133.57, 130.03, 129.24, 126.66, 122.60, 121.76, 119.34, 118.73, 117.46, 115.13, 111.70, 111.55, 84.23, 67.45, 56.20, 56.02,, 38.58, 27.67. HR-MS (ESI) *m/z*: calcd for C_26_H_25_O_6_N_2_ [M+H]^+^, 461.1707; found, 461.1704.

*N-(2,3-dimethoxy-8-oxo-5,13a-dihydro-6H,8H-benzo[5,6][1,3]oxazino[2,3-a]isoquinolin-10-yl)-3-(dimethylamino)propanamide (****3i****)* ^1^H NMR (400 MHz, DMSO-*d*_6_) δ 10.62 (s, 2H), 8.20 (d, *J* = 2.7 Hz, 1H), 7.76 (dd, *J* = 8.9, 2.7 Hz, 1H), 7.13 (d, *J* = 8.8 Hz, 1H), 7.08 (s, 1H), 6.90 (s, 1H), 6.32 (s, 1H), 4.41 – 4.30 (m, 1H), 3.80 (d, *J* = 1.9 Hz, 6H), 3.21 (ddd, *J* = 12.8, 10.9, 4.0 Hz, 1H), 2.95 – 2.78 (m, 4H), 2.75 (s, 6H). ^13^C NMR (100 MHz, DMSO-*d*_6_) δ 168.72, 162.40, 153.36, 149.86, 148.18, 134.37, 129.23, 125.84, 122.69, 118.76, 118.53, 117.40, 111.76, 111.62, 84.22, 56.24, 56.05, 53.44, 43.17, 38.55, 31.97, 27.72. HR-MS (ESI) *m/z*: calcd for C_23_H_28_O_5_N_3_ [M+H]^+^, 426.2023; found, 426.2024.

*Methyl(2,3-dimethoxy-8-oxo-5,13a-dihydro-6H,8H-benzo[5,6][1,3]oxazino[2,3-a]isoquinolin-10-yl)carbamate (****3j****)* ^1^H NMR (400 MHz, DMSO-*d*_6_) δ 9.72 (s, 1H), 7.99 (d, *J* = 2.7 Hz, 1H), 7.60 (dd, *J* = 8.8, 2.7 Hz, 1H), 7.15 – 7.03 (m, 2H), 6.89 (s, 1H), 6.30 (s, 1H), 4.36 (dt, *J* = 12.8, 4.0 Hz, 1H), 3.79 (d, *J* = 2.2 Hz, 6H), 3.68 (s, 3H), 3.21 (ddd, *J* = 12.7, 10.8, 3.9 Hz, 1H), 2.89 (ddd, *J* = 15.7, 10.9, 4.8 Hz, 1H), 2.83 – 2.73 (m, 1H). ^13^C NMR (100 MHz, DMSO-*d*_6_) δ 162.44, 154.61, 152.88, 149.83, 148.16, 134.47, 129.22, 124.97, 122.71, 118.87, 117.51, 111.72, 111.58, 84.18, 56.21, 56.03, 52.20, 38.54, 27.71. HR-MS (ESI) *m/z*: calcd for C_20_H_21_O_6_N_2_ [M+H]^+^, 385.1394; found, 385.1385.

*1-(3-chlorophenyl)-3-(2,3-dimethoxy-8-oxo-5,13a-dihydro-6H,8H-benzo[5,6][1,3]oxazino[2,3-a]isoquinolin-10-yl)urea (****3k****)* ^1^H NMR (400 MHz, DMSO-*d*_6_) δ 8.88 (d, *J* = 6.5 Hz, 2H), 8.00 (d, *J* = 2.7 Hz, 1H), 7.71 (q, *J* = 1.5 Hz, 1H), 7.58 (dd, *J* = 8.8, 2.7 Hz, 1H), 7.33 – 7.24 (m, 2H), 7.15 – 7.07 (m, 2H), 7.06 – 6.98 (m, 1H), 6.90 (s, 1H), 6.32 (s, 1H), 4.42 – 4.30 (m, 1H), 3.23 (td, *J* = 12.4, 11.8, 3.9 Hz, 1H), 2.94 – 2.75 (m, 2H). ^13^C NMR (100 MHz, DMSO-*d*_6_) δ 162.50, 152.99, 152.75, 149.83, 148.16, 141.68, 134.70, 133.64, 130.88, 129.24, 125.53, 122.74, 122.00, 118.87, 118.14, 117.79, 117.50, 117.23, 111.72, 111.59, 84.19, 56.22, 56.03, 38.56, 27.72. HR-MS (ESI) *m/z*: calcd for C_25_H_23_O_5_N_3_Cl [M+H]^+^, 480.1321; found, 480.1363.

- 1. *General procedure for the synthesis of compounds* ***4a****-****4j***

To a solution of 6,7-dimethoxy-3,4-dihydroisoquinoline (1.0 equiv) and 2-hydroxy-5-(methoxycarbonyl)benzoic acid (1.1 equiv) in dichloromethane was added 1-(3-dimethylaminopropyl)-3-ethylcarbodiimide hydrochloride (2.0 equiv). The mixture was stirred at room temperature for 8 h. After completion of the reaction, the mixture was diluted with dichloromethane. Subsequently, the mixture was washed with a saturated solution of NaHCO_3_, H_2_O, and a saturated solution of NaCl. The dichloromethane phase was dried over anhydrous Na_2_SO_4_, filtered and concentrated under vacuum. The crude product was purified by silica gel column chromatography to obtain **8**, yield 45.2%. ^1^H NMR (400 MHz, DMSO-*d*_6_) δ 8.44 (d, *J* = 2.2 Hz, 1H), 8.13 (dd, *J* = 8.6, 2.3 Hz, 1H), 7.29 (d, *J* = 8.6 Hz, 1H), 7.12 (s, 1H), 6.91 (s, 1H), 6.49 (s, 1H), 4.40 (ddd, *J* = 12.8, 4.8, 3.0 Hz, 1H), 3.88 (s, 3H), 3.80 (d, *J* = 2.5 Hz, 6H), 3.28 – 3.13 (m, 1H), 2.91 (ddd, *J* = 15.9, 11.1, 4.9 Hz, 1H), 2.84 – 2.76 (m, 1H).

To a solution of **8** (1.0 equiv) in MeOH and H_2_O (3:1) was added LiOH (2.0 equiv). The mixture was stirred at room temperature for 12 h. After completion of the reaction, the mixture was filtered and evaporated under reduced pressure. The crude product was purified by silica gel column chromatography to obtain **9**, yield 82.4%. ^1^H NMR (400 MHz, DMSO-*d*_6_) δ 13.07 (s, 1H), 8.43 (d, *J* = 2.2 Hz, 1H), 8.10 (dd, *J* = 8.6, 2.2 Hz, 1H), 7.26 (d, *J* = 8.6 Hz, 1H), 7.12 (s, 1H), 6.91 (s, 1H), 6.47 (s, 1H), 4.44 – 4.33 (m, 1H), 3.80 (d, *J* = 2.3 Hz, 7H), 3.28 – 3.16 (m, 2H), 2.91 (ddd, *J* = 15.9, 11.0, 4.8 Hz, 1H), 2.85 – 2.77 (m, 1H).

To a solution of **9** (1.0 equiv) with different amine (0.9 equiv) in dichloromethane was added 2-(7-azabenzotriazol-1-yl)-N,N,N',N'-tetramethyluronium hexafluorophosphate (1.5 equiv) and N,N-Diisopropylethylamine (3.0 equiv). The mixture was stirred at room temperature for 12 h. After completion of the reaction, the mixture was diluted with dichloromethane. Subsequently, the mixture was washed with a saturated solution of NaHCO_3_, H_2_O, and a saturated solution of NaCl. The dichloromethane phase was dried over anhydrous Na_2_SO_4_, filtered and concentrated under vacuum. The crude product was purified by silica gel column chromatography to obtain **4a**-**4j.**

*2,3-dimethoxy-8-oxo-N-phenyl-5,13a-dihydro-6H,8H-benzo[5,6][1,3]oxazino[2,3-a]isoquinoline-10-carboxamide (****4a****)* ^1^H NMR (400 MHz, DMSO-*d*_6_) δ 10.40 (s, 1H), 8.57 (d, *J* = 2.3 Hz, 1H), 8.20 (dd, *J* = 8.6, 2.3 Hz, 1H), 7.84 – 7.73 (m, 2H), 7.41 – 7.26 (m, 3H), 7.16 – 7.06 (m, 2H), 6.92 (s, 1H), 6.48 (s, 1H), 4.43 (ddd, *J* = 12.7, 4.7, 3.1 Hz, 1H), 3.81 (s, 6H), 3.30 – 3.20 (m, 1H), 2.93 (ddd, *J* = 15.8, 11.1, 4.8 Hz, 1H), 2.86 – 2.75 (m, 1H). ^13^C NMR (100 MHz, DMSO-*d*_6_) δ 164.73, 162.18, 159.94, 149.97, 148.19, 139.39, 134.24, 129.40, 129.35, 129.12, 128.30, 124.35, 122.17, 121.08, 118.45, 117.48, 111.74, 111.63, 84.53, 56.22, 56.04, 38.74, 27.60. HR-MS (ESI) *m/z*: calcd for C_25_H_23_O_5_N_2_ [M+H]^+^, 431.1601; found, 431.1594.

*2,3-dimethoxy-8-oxo-N-propyl-5,13a-dihydro-6H,8H-benzo[5,6][1,3]oxazino[2,3-a]isoquinoline-10-carboxamide (****4b****)* ^1^H NMR (400 MHz, DMSO-*d*_6_) δ 8.61 (t, *J* = 5.6 Hz, 1H), 8.42 (d, *J* = 2.3 Hz, 1H), 8.06 (dd, *J* = 8.6, 2.3 Hz, 1H), 7.23 (d, *J* = 8.6 Hz, 1H), 7.12 (s, 1H), 6.91 (s, 1H), 6.43 (s, 1H), 4.40 (ddd, *J* = 12.8, 4.8, 3.2 Hz, 1H), 3.80 (d, *J* = 1.8 Hz, 7H), 3.29 – 3.11 (m, 3H), 2.91 (ddd, *J* = 15.8, 11.0, 4.8 Hz, 1H), 2.80 (dt, *J* = 15.8, 3.7 Hz, 1H), 1.54 (p, *J* = 7.3 Hz, 2H), 0.90 (t, *J* = 7.4 Hz, 3H). ^13^C NMR (100 MHz, DMSO-*d*_6_) δ 165.24, 162.22, 159.53, 149.97, 148.22, 133.71, 129.32, 127.64, 122.31, 118.40, 117.23, 111.79, 111.67, 84.45, 56.25, 56.06, 41.54, 38.67, 27.67, 22.83, 11.97. HR-MS (ESI) *m/z*: calcd for C_22_H_25_O_5_N_2_ [M+H]^+^, 397.1758; found, 397.1752.

*N-(2-(dimethylamino)ethyl)-2,3-dimethoxy-8-oxo-5,13a-dihydro-6H,8H-benzo[5,6][1,3]oxazino[2,3-a]isoquinoline-10-carboxamide (****4c****)* ^1^H NMR (400 MHz, DMSO-*d*_6_) δ 8.56 (t, *J* = 5.6 Hz, 1H), 8.41 (d, *J* = 2.3 Hz, 1H), 8.05 (dd, *J* = 8.6, 2.3 Hz, 1H), 7.23 (d, *J* = 8.6 Hz, 1H), 7.11 (s, 1H), 6.91 (s, 1H), 6.43 (s, 1H), 4.44 – 4.35 (m, 1H), 3.80 (d, *J* = 1.8 Hz, 6H), 3.37 (q, *J* = 6.4 Hz, 2H), 3.24 (ddd, *J* = 12.7, 11.1, 4.0 Hz, 1H), 2.96 – 2.85 (m, 1H), 2.85 – 2.75 (m, 1H), 2.43 (t, *J* = 6.8 Hz, 2H), 2.20 (s, 6H). ^13^C NMR (100 MHz, DMSO-*d*_6_) δ 165.51, 162.24, 159.60, 149.94, 148.18, 133.74, 129.32, 129.09, 127.66, 122.21, 118.36, 117.32, 111.72, 111.61, 84.45, 58.43, 56.21, 56.03, 45.50, 38.69, 37.72, 27.60. HR-MS (ESI) *m/z*: calcd for C_23_H_28_O_5_N_3_ [M+H]^+^, 426.2023; found, 426.2033.

*N-(2-(diethylamino)ethyl)-2,3-dimethoxy-8-oxo-5,13a-dihydro-6H,8H-benzo[5,6][1,3]oxazino[2,3-a]isoquinoline-10-carboxamide (****4d****)* ^1^H NMR (400 MHz, DMSO-*d*_6_) δ 8.54 (t, *J* = 5.7 Hz, 1H), 8.40 (d, *J* = 2.2 Hz, 1H), 8.04 (dd, *J* = 8.6, 2.3 Hz, 1H), 7.23 (d, *J* = 8.6 Hz, 1H), 7.11 (s, 1H), 6.91 (s, 1H), 6.43 (s, 1H), 4.40 (ddd, *J* = 12.8, 4.8, 3.2 Hz, 1H), 3.28 – 3.18 (m, 1H), 2.89 (dd, *J* = 10.9, 4.8 Hz, 1H), 2.85 – 2.74 (m, 1H), 2.60 – 2.51 (m, 4H), 0.97 (t, *J* = 7.1 Hz, 6H). ^13^C NMR (100 MHz, DMSO-*d*_6_) δ 165.25, 162.21, 159.56, 149.97, 148.21, 133.67, 129.32, 129.24, 127.55, 122.28, 118.40, 117.29, 111.77, 111.67, 109.98, 84.46, 56.23, 56.05, 51.86, 47.28, 38.68, 38.04, 27.65, 12.37. HR-MS (ESI) *m/z*: calcd for C_25_H_32_O_5_N_3_ [M+H]^+^, 454.2336; found, 454.2340.

*N-(2-(dibutylamino)ethyl)-2,3-dimethoxy-8-oxo-5,13a-dihydro-6H,8H-benzo[5,6][1,3]oxazino[2,3-a]isoquinoline-10-carboxamide (****4e****)* ^1^H NMR (400 MHz, DMSO-*d*_6_) δ 8.50 (t, *J* = 5.7 Hz, 1H), 8.40 (d, *J* = 2.3 Hz, 1H), 8.04 (dd, *J* = 8.6, 2.3 Hz, 1H), 7.23 (d, *J* = 8.5 Hz, 1H), 7.12 (s, 1H), 6.91 (s, 1H), 6.43 (s, 1H), 4.40 (dt, *J* = 12.8, 3.8 Hz, 1H), 3.80 (d, *J* = 2.0 Hz, 6H), 3.30 – 3.14 (m, 3H), 2.91 (ddd, *J* = 15.8, 11.0, 4.7 Hz, 1H), 2.80 (dt, *J* = 15.8, 3.6 Hz, 1H), 2.56 (t, *J* = 7.2 Hz, 2H), 2.42 (t, *J* = 7.2 Hz, 4H), 1.41 – 1.35 (m, 4H), 1.31 – 1.25 (m, 4H), 0.87 (t, *J* = 7.3 Hz, 6H). ^13^C NMR (101 MHz, DMSO-*d*_6_) δ 165.31, 162.21, 159.55, 149.95, 148.20, 133.64, 129.32, 129.22, 127.53, 122.25, 118.37, 117.29, 111.74, 111.64, 84.45, 56.21, 56.04, 53.98, 52.95, 38.68, 37.98, 29.42, 27.63, 20.48, 14.41. HR-MS (ESI) *m/z*: calcd for C_29_H_40_O_5_N_3_ [M+H]^+^, 510.2962; found, 510.2962.

*2,3-dimethoxy-8-oxo-N-(2-(pyrrolidin-1-yl)ethyl)-5,13a-dihydro-6H,8H-benzo[5,6][1,3]oxazino[2,3-a]isoquinoline-10-carboxamide (****4f****)* ^1^H NMR (400 MHz, CDCl_3_) δ 8.62 (d, *J* = 2.2 Hz, 1H), 8.49 (s, 1H), 8.23 (dd, *J* = 8.6, 2.3 Hz, 1H), 7.12 (d, *J* = 8.6 Hz, 1H), 7.01 (s, 1H), 6.71 (s, 1H), 6.23 (s, 1H), 4.56 (dt, *J* = 12.9, 4.2 Hz, 1H), 3.93 (d, *J* = 10.8 Hz, 6H), 3.89 (d, *J* = 5.2 Hz, 2H), 3.40 – 3.22 (m, 7H), 3.03 (ddd, *J* = 15.8, 11.0, 4.8 Hz, 1H), 2.77 (d, *J* = 15.8 Hz, 1H), 2.13 (p, *J* = 3.5 Hz, 4H). ^13^C NMR (100 MHz, CDCl_3_) δ 166.71, 162.35, 159.88, 149.94, 148.37, 133.42, 128.93, 128.05, 121.85, 118.39, 116.91, 110.86, 110.48, 84.33, 56.19, 56.03, 54.64, 38.51, 36.37, 29.71, 27.89, 23.36. HR-MS (ESI) *m/z*: calcd for C_25_H_30_O_5_N_3_ [M+H]^+^, 452.2180; found, 452.2174.

*2,3-dimethoxy-8-oxo-N-(2-(piperidin-1-yl)ethyl)-5,13a-dihydro-6H,8H-benzo[5,6][1,3]oxazino[2,3-a]isoquinoline-10-carboxamide (****4g****)* ^1^H NMR (400 MHz, CDCl_3_) δ 8.28 (d, *J* = 2.3 Hz, 1H), 7.89 (dd, *J* = 8.5, 2.2 Hz, 1H), 7.76 (s, 1H), 6.99 (d, *J* = 8.6 Hz, 1H), 6.93 (s, 1H), 6.70 (s, 1H), 6.11 (s, 1H), 4.53 – 4.42 (m, 1H), 3.90 (d, *J* = 4.7 Hz, 6H), 3.84 (s, 2H), 3.45 (s, 2H), 3.28 – 3.19 (m, 1H), 3.04 – 2.94 (m, 1H), 2.75 (d, *J* = 15.9 Hz, 1H), 1.82 (d, *J* = 93.0 Hz, 8H), 1.26 (s, 2H). ^13^C NMR (100 MHz, CDCl_3_) δ 169.43, 162.26, 160.04, 150.00, 148.32, 133.44, 128.84, 128.04, 126.95, 121.37, 117.81, 117.14, 110.93, 110.47, 84.25, 58.76, 56.17, 56.04, 54.23, 47.34, 38.70, 35.96, 29.72, 27.63, 23.61, 21.46, 8.68. HR-MS (ESI) *m/z*: calcd for C_26_H_32_O_5_N_3_ [M+H]^+^, 466.2336; found, 466.2335.

*2,3-dimethoxy-N-(2-morpholinoethyl)-8-oxo-5,13a-dihydro-6H,8H-benzo[5,6][1,3]oxazino[2,3-a]isoquinoline-10-carboxamide (****4h****)* ^1^H NMR (400 MHz, DMSO-*d*_6_) δ 8.58 (t, *J* = 5.6 Hz, 1H), 8.41 (d, *J* = 2.3 Hz, 1H), 8.05 (dd, *J* = 8.6, 2.3 Hz, 1H), 7.24 (d, *J* = 8.6 Hz, 1H), 7.11 (s, 1H), 6.91 (s, 1H), 6.43 (s, 1H), 4.40 (ddd, *J* = 12.7, 4.6, 3.1 Hz, 1H), 3.80 (d, *J* = 1.9 Hz, 6H), 3.57 (t, *J* = 4.6 Hz, 4H), 3.39 (q, *J* = 6.6 Hz, 2H), 3.24 (ddd, *J* = 12.8, 11.0, 3.9 Hz, 1H), 2.96 – 2.86 (m, 1H), 2.84 – 2.76 (m, 1H), 2.49 – 2.40 (m, 6H). ^13^C NMR (100 MHz, DMSO-*d*_6_) δ 165.50, 162.23, 159.60, 149.95, 148.19, 133.72, 129.33, 129.13, 127.63, 122.22, 118.38, 117.34, 111.73, 84.45, 66.61, 57.75, 56.21, 56.04, 53.71, 38.70, 37.00, 27.61. HR-MS (ESI) *m/z*: calcd for C_25_H_30_O_6_N_3_ [M+H]^+^, 468.2129; found, 468.2124.

*2,3-dimethoxy-N-(2-(4-methylpiperazin-1-yl)ethyl)-8-oxo-5,13a-dihydro-6H,8H-benzo[5,6][1,3]oxazino[2,3-a]isoquinoline-10-carboxamide (****4i****)* ^1^H NMR (400 MHz, CDCl_3_) δ 8.37 (s, 1H), 8.11 (d, *J* = 8.7 Hz, 1H), 7.16 (d, *J* = 8.6 Hz, 1H), 7.02 (s, 2H), 6.72 (s, 1H), 6.27 (s, 1H), 4.59 (d, *J* = 12.8 Hz, 1H), 3.94 (d, *J* = 10.5 Hz, 6H), 3.60 (d, *J* = 5.9 Hz, 2H), 3.34 (t, *J* = 11.7 Hz, 1H), 3.16 (s, 1H), 3.04 (d, *J* = 12.2 Hz, 1H), 2.86 – 2.61 (m, 10H), 2.45 (s, 3H). ^13^C NMR (100 MHz, DMSO-*d*_6_) δ 165.65, 162.24, 159.62, 149.93, 148.16, 133.73, 129.32, 129.03, 127.68, 122.17, 118.35, 117.35, 111.71, 111.58, 84.44, 56.73, 56.20, 56.03, 53.96, 51.44, 44.45, 38.70, 37.07, 27.58.HR-MS (ESI) *m/z*: calcd for C_26_H_33_O_5_N_4_ [M+H]^+^, 481.2445; found, 481.2450.

*2,3-dimethoxy-10-(4-methylpiperazine-1-carbonyl)-5,13a-dihydro-6H,8H-benzo[5,6][1,3]oxazino[2,3-a]isoquinolin-8-one (****4j****)* ^1^H NMR (400 MHz, DMSO-*d*_6_) δ 7.84 (d, *J* = 2.1 Hz, 1H), 7.60 (dd, *J* = 8.4, 2.2 Hz, 1H), 7.22 (d, *J* = 8.4 Hz, 1H), 7.11 (s, 1H), 6.91 (s, 1H), 6.43 (s, 1H), 5.76 (s, 1H), 4.38 (dt, *J* = 12.9, 4.3 Hz, 1H), 3.80 (d, *J* = 2.3 Hz, 6H), 3.49 (s, 4H), 3.27 – 3.11 (m, 1H), 2.95 – 2.84 (m, 1H), 2.84 – 2.75 (m, 1H), 2.36 (s, 4H), 2.22 (s, 3H). ^13^C NMR (100 MHz, DMSO-*d*_6_) δ 168.57, 162.14, 158.36, 149.94, 148.18, 133.74, 130.27, 129.33, 127.27, 122.18, 118.48, 117.56, 111.72, 111.64, 84.42, 56.20, 56.03, 45.80, 38.69, 27.59. HR-MS (ESI) *m/z*: calcd for C_24_H_28_O_5_N_3_ [M+H]^+^, 438.2023; found, 438.2022.

**2. ^1^H NMR, ^13^C NMR and HR-MS spectra of 3a-3k and 4a-4j**

**^1^H NMR spectra of 3a**

**^13^C NMR spectra of 3a**

**HR-MS spectra of 3a**

**^1^H NMR spectra of 3b**

**^13^C NMR spectra of 3b**

**HR-MS spectra of 3b**

**^1^H NMR spectra of 3c**

**^13^C NMR spectra of 3c**

**HR-MS spectra of 3c**

**^1^H NMR spectra of 3d**

**^13^C NMR spectra of 3d**

**HR-MS spectra of 3d**

**^1^H NMR spectra of 3e**

**^13^C NMR spectra of 3e**

**HR-MS spectra of 3e**

**^1^H NMR spectra of 3f**

**^13^C NMR spectra of 3f**

**HR-MS spectra of 3f**

**^1^H NMR spectra of 3g**

**^13^C NMR spectra of 3g**

**HR-MS spectra of 3g**

**^1^H NMR spectra of 3h**

**^13^C NMR spectra of 3h**

**HR-MS spectra of 3h**

**^1^H NMR spectra of 3i**

**^13^C NMR spectra of 3i**

**HR-MS spectra of 3i**

**^1^H NMR spectra of 3j**

**^13^C NMR spectra of 3j**

**HR-MS spectra of 3j**

**^1^H NMR spectra of 3k**

**^13^C NMR spectra of 3k**

**HR-MS spectra of 3k**

**^1^H NMR spectra of 4a**

**^13^C NMR spectra of 4a**

**HR-MS spectra of 4a**

**^1^H NMR spectra of 4b**

**^13^C NMR spectra of 4b**

**HR-MS spectra of 4b**

**^1^H NMR spectra of 4c**

**^13^C NMR spectra of 4c**

**HR-MS spectra of 4c**

**^1^H NMR spectra of 4d**

**^13^C NMR spectra of 4d**

**HR-MS spectra of 4d**

**^1^H NMR spectra of 4e**

**^13^C NMR spectra of 4e**

**HR-MS spectra of 4e**

**^1^H NMR spectra of 4f**

**^13^C NMR spectra of 4f**

**HR-MS spectra of 4f**

**^1^H NMR spectra of 4g**

**^13^C NMR spectra of 4g**

**HR-MS spectra of 4g**

**^1^H NMR spectra of 4h**

**^13^C NMR spectra of 4h**

**HR-MS spectra of 4h**

**^1^H NMR spectra of 4i**

**^13^C NMR spectra of 4i**

**HR-MS spectra of 4i**

**^1^H NMR spectra of 4j**

**^13^C NMR spectra of 4j**

**HR-MS spectra of 4j**
